# Global long term daily 1 km surface soil moisture dataset with physics informed machine learning

**DOI:** 10.1038/s41597-023-02011-7

**Published:** 2023-02-17

**Authors:** Qianqian Han, Yijian Zeng, Lijie Zhang, Chao Wang, Egor Prikaziuk, Zhenguo Niu, Bob Su

**Affiliations:** 1 Faculty of Geo-Information Science and Earth Observation (ITC), University of Twente, 7514 AE Enschede, The Netherlands; 2 Research Center Jülich, Institute of Bio- and Geosciences: Agrosphere (IBG-3), 52428 Jülich, Germany; 3 Department of Earth, Marine and Environmental Sciences, University of North Carolina, Chapel Hill, NC USA; 4 State Key Laboratory of Remote Sensing Science, Aerospace Information Research Institute, Chinese Academy of Sciences, Beijing, 100101 China; 5 Key Laboratory of Subsurface Hydrology and Ecological Effect in Arid Region of Ministry of Education, School of Water and Environment, Chang’an University, Xi’an, 710054 China

**Keywords:** Hydrology, Environmental sciences

## Abstract

Although soil moisture is a key factor of hydrologic and climate applications, global continuous high resolution soil moisture datasets are still limited. Here we use physics-informed machine learning to generate a global, long-term, spatially continuous high resolution dataset of surface soil moisture, using International Soil Moisture Network (ISMN), remote sensing and meteorological data, guided with the knowledge of physical processes impacting soil moisture dynamics. Global Surface Soil Moisture (GSSM1 km) provides surface soil moisture (0–5 cm) at 1 km spatial and daily temporal resolution over the period 2000–2020. The performance of the GSSM1 km dataset is evaluated with testing and validation datasets, and via inter-comparisons with existing soil moisture products. The root mean square error of GSSM1 km in testing set is 0.05 cm^3^/cm^3^, and correlation coefficient is 0.9. In terms of the feature importance, Antecedent Precipitation Evaporation Index (APEI) is the most important significant predictor among 18 predictors, followed by evaporation and longitude. GSSM1 km product can support the investigation of large-scale climate extremes and long-term trend analysis.

## Background & Summary

Surface soil moisture (SSM) is a source of water for the atmosphere through processes leading to evapotranspiration from land^1–3^. SSM has impacts on climate processes by influencing the partitioning of the incoming energy in the latent and sensible heat fluxes and controlling the partitioning of precipitation into runoff, evapotranspiration, and infiltration^2,3^. Therefore, a global high resolution, long-term, and spatiotemporally consistent SSM dataset is necessary for understanding the processes between the land surface and atmosphere, and is useful for numerous applications, e.g. flood and drought monitoring, irrigation scheduling, and agricultural management.

Although SSM has such high importance from many perspectives, there is still a paucity of global-scale long-term high resolution SSM datasets with acceptable precision and accuracy. There are three main sources of SSM^2,4–6^: *in-situ* soil moisture, satellite observations, and soil moisture products from either Machine Learning (ML) algorithms or Land Surface Model (LSM)^2,7^. The *in-situ* observations provide continuous observations from different soil depths at the point scale. Satellite observations allow the retrieval of soil moisture at a global scale. However, satellite retrievals have spatiotemporal gaps, due to revisit time, land surface states, or complex topography^1^. LSM can be used to produce global soil moisture but there are big differences among different products due to different and uncertain parameterizations^1,5,8^. As a result, each type of soil moisture has its own advantages and limitations. There are soil moisture datasets at the global scale from satellites, e.g. AMSR2, ASCAT, Sentinel-1, SMAP, SMOS, ESA-CCI, and from LSM, e.g. ERA-5, GLDAS^7^. These products differ in terms of spatiotemporal resolution, coverage, and data sources. Among these products, SMAP presents a better performance and has the highest spatial resolution (1–36 km) but it has a shorter time span (from 2015 until now)^9^.

ML makes it possible to produce high resolution soil moisture datasets by learning the relationship between the *in-situ* soil moisture and its driving factors at a global scale^1^. Several soil moisture products based on ML have been presented, NNsm with 36 km resolution at a global scale (daily, 2002–2019) based on Artificial neural networks (ANN)^10^, SoMo.ml with 0.25° spatial resolution at a global scale (daily, 2000–2019) based on Long Short-Term Memory neural network (LSTM)^1^, and a soil moisture product with 0.25° resolution at global scale (daily, 2000–2018) based on Random Forest (RF)^2^. These datasets provide us the possibility to do soil moisture related research, indicating that ML is a promising tool to predict soil moisture. Nevertheless, there is a lack of high spatial-temporal resolution (e.g. 1 km daily) soil moisture with high precision and accuracy.

This study aims to present a global long-term daily 1 km surface soil moisture dataset through physics-informed RF. Namely, we used RF to build a soil moisture prediction model, with related meteorological forcings and static features obtained from both satellite and reanalysis datasets, while guided by the physical understanding of processes impacting soil moisture dynamics. The produced Global Surface Soil Moisture (GSSM1 km) dataset has a temporal coverage of 21 years (2000–2020) with a daily 1 km resolution.

## Methods

### Physics-informed RF and predictor variables

**Fig. 1:**
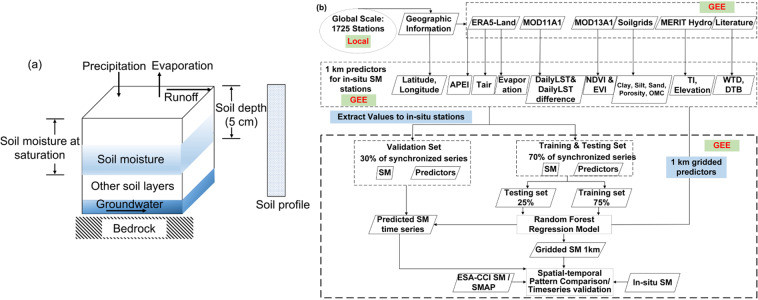
(**a**) Conceptual diagram; (**b**) Schematic overview of the methodology.

From the physical process perspective (Fig. 1a), there are many land surface features affecting SSM in the land-atmosphere interaction^6^. In this study, 18 predictors were used to predict SSM. The data source of them is shown in Table 1 and the detailed processing steps are provided in supplementary materials.

**Table 1:** Predictors used for the RF model (for more details about source data and data processing see supplementary materials section 1: Satellite and reanalysis data, and section 2: Data processing).

	Predictors	source	Spatial resolution	Temporal resolution	Time span	Unit of predictors
Dynamic	APEI	ERA5Land	11 km	Hourly	1981-1-1 to now	mm
	Tair		11 km	Hourly		°C
	Evaporation		11 km	Hourly		mm
	Daily LST	MOD11A1	1 km	Daily	2000-2-24 to now	°C
	Daily LST Diff		1 km	Daily		°C
	NDVI	MOD13A2	1 km	16-day	2000-2-18 to now	/
	EVI		1 km	16-day		/
Static	Longitude	/	/	/	/	/
	Latitude	/	/	/	/	
	Elevation	MERIT Hydro	92 m		/	m
	TI		92 m	/	/	/
	Soil Texture (sand, silt, clay fraction)	SoilGrids	250 m	/	/	%
	Porosity		250 m	/	/	%
	OMC		250 m	/	/	%
	WTD	Ying Fan^36^	1 km	/	/	m
	DTB	Wei Shangguan^39^	1 km	/	/	m

The spatial and temporal changes of soil water storage depend on the variability of precipitation (*P*), evaporation (*Evapo*), and runoff (*R*)^11,12^ (Fig. 1a). Precipitation has a moderate to strong positive correlation with soil moisture^13^. Evaporation is the process that water – originating from a wide range of sources – is transferred from the soil compartment and/or vegetation to the atmosphere. Evaporation directly connects with soil moisture since soil moisture that can potentially evaporate is usually related to water contained in the upper 1–2 m of a soil profile^14^. The cumulative water balance, calculated as the surplus between precipitation and evapotranspiration & runoff (i.e., *P*-*Evapo*-*R*), in previous days influences the soil moisture in the current day^15,16^. Therefore, Antecedent Precipitation Evaporation Index (APEI) is used in this study which indicates the time-weighted summation of precipitation and evapotranspiration over a specific time window^16,17^. APEI can reflect some soil moisture characteristics caused by meteorological elements, such as precipitation and evapotranspiration. The historical precipitation and evapotranspiration influence the soil moisture in a weakening effect along the reverse time axis, which means the most recent precipitation and evapotranspiration event has a higher impact on the current soil moisture. The detailed calculation of APEI can be found in section 2.1 in supplementary materials.

Land Surface Temperature (LST) is the radiative skin temperature of the land driven by solar radiation, which measures the emission of thermal radiance from the land surface where the incoming solar energy interacts with and heats the ground surface or the canopy in vegetated areas^18^. After the solar energy is absorbed by the ground, the ground transfers part of the heat to the air through radiation, conduction, and convection, which is the main source of heat in the air. LST and air temperature are intrinsically distinct yet often strongly related because the temperature between them determines the sensible heat flux, and their correlation arises from the surface energy balance^19,20^. There is a negative feedback between soil moisture and air temperature and LST^2,21^. Furthermore, the daily LST difference is strongly related to the thermal inertia of soil, while thermal inertia increases with soil moisture^22,23^.

The vegetation index is the reflectance transformation of two or more spectral bands from satellite images. For example, the Normalized Difference Vegetation Index (NDVI) is one of the most used vegetation indices, representing the greenness of the vegetation condition, and is considered as a conservative water stress index^24^. Plenty of research has been done on retrieving SSM with the help of vegetation indices. Temperature/Vegetation Dryness Index has a strong negative relationship with SSM, and SSM has been often estimated using LST, albedo, and NDVI^25,26^. In addition, the Enhanced Vegetation Index (EVI) is also commonly used to improve the sensitivity of SSM estimation at high vegetation-covered areas^27^.

Besides the above dynamic predictors, static soil physical properties including soil texture, porosity, and organic matter content (OMC) also affect soil moisture. Soil texture refers to the composition of the soil in terms of the proportion of small, medium, and large particles (clay, silt, and sand, respectively) in a specific soil mass^28^. Soil porosity refers to the space between soil particles, which consists of various amounts of water and air^28^. Water-holding capacity is controlled primarily by soil texture and organic matter. Soil with smaller particles (silt and clay) has a larger surface area than those with larger sand particles, and a large surface area allows soil to hold more water. Organic matter content (OMC) also influences water-holding capacity. As the content increases, the water-holding capacity increases because of the affinity organic matter has for water.

A study in Switzerland shows elevation determines SSM dynamics, but the relation between SSM and elevation is non-linear^29^. The SSM regularly increases with an increasing elevation below 2000 m a.s.l (above sea level), and then decreases with elevation above 2000 m a.s.l^29^. This tipping point also corresponds to a clear shift in the SSM regime. Below 2000 m a.s.l, the maximum SSM is recorded in winter and the minimum in summer, while above this threshold it occurs the opposite (maximum SSM in summer and minimum in winter)^29^.

Topography is an important determinant of SSM distribution, and plenty of indices have been used to assess SSM spatial variability^30^. The most frequently used index, the topographic index (TI), is based on the topography of landscapes and was first introduced in TOPography based hydrological MODEL (TOP-MODEL) to generate the patterns of runoff-contributing areas governed by a saturation runoff generation process in landscapes^30–33^. TI quantifies the trends of soil moisture distribution, which is affected by topography^30^.

The latitude determines the solar radiation and temperature and the longitude relates to the closeness to the oceans (moisture and temperature), atmospheric circulation, and the amount of precipitation. The incoming solar radiation plays an important role in determining SSM variability^34^. Solar radiation and temperature are the thermal (radiation and sensible heat energy) sources that cause water to evaporate from the earth’s surface^35^.

The groundwater table is an undulating surface between the oxygenated soils and the water-saturated aquifers below^36^. Groundwater may have a small effect on soil moisture in areas with a deep water table depth (WTD), but it can act as a SSM source and have substantial effects in areas where the water table depth is shallow by sustaining river base-flow and root-zone SSM in the absence of rain^36,37^. The water table depth distribution in these areas creates an additional spatial heterogeneity, similar to that created by variations in topography, surface vegetation, and soil properties, and is critical for regional processes affecting spatial variations of SSM^38^.

Bedrock is either exposed at the earth surface or buried under soil and regolith, which is a key parameter of interest because it restricts root penetration of plants^39,40^. Depth to bedrock (DTB) is considered as the lower boundary in land surface modeling, which controls the energy, water, and carbon cycle^39^. DTB is equivalent to the total thickness of the solum and weathered rocks^40^.

### *In-situ* soil moisture data

*In-situ* soil moisture data are provided from the International Soil Moisture Network (ISMN) website. The ISMN was initialized to collect the *in-situ* soil moisture into an open-access database  in 2009. By the end of 2019, the database consisted of 2443 stations from 58 networks around the world, and ISMN is still growing.

The *in-situ* data were collected from different organizations and groups. There is no standard protocol for the soil moisture collection strategy, massive diversity has been seen between the data from various networks, e.g. sensor types, sensor installation depths, and temporal measurement steps. For all these reasons, extensive efforts have been made to harmonize the *in-situ* soil moisture through a prime data quality control system, and to improve the reliability of the *in-situ* data^41^. Besides, the observation time has been converted from local time to Coordinated Universal Time (UTC), and the temporal resolution was also harmonized into hourly intervals for convenience, the time span is 2000 to 2018.

### Machine learning and prediction

We trained a Random Forest (RF) regression model on the Google earth engine (GEE) to generate the GSSM1 km dataset. GEE is a cloud-based platform for planetary-scale geospatial analysis that brings Google’s massive computational capabilities to serve a variety of high-impact societal issues including deforestation, drought, disaster, disease, food security, water management, climate monitoring, and environmental protection^42,43^. Random Forest (RF) regression is an ensemble learning method that outputs a result based on the mean of the many individual training models (trees). RF follows the Bootstrap Aggregation (Bagging) strategies, i.e. random sampling with replacement^44^.

The RF model was trained to learn the relationship between the 18 predictors and soil moisture. All 18 predictors were synchronized based on the temporal coverage of *in-situ* data time-series of each ISMN station. We used the following strategy for data split: First, divide the predictors and SSM time series into training & testing set (70%) and validation set (30%). For example, assuming the data were recorded from 1 January 2000 to 31 December 2019, the training & testing set consists of the first 70% data (14 years, from 2000 to 2013), and the validation set consists of the last 30% data (6 years, from 2014 to 2019). Second, split the training & testing set into two parts (e.g., training set and testing set) randomly with the proportion of 75% and 25% (in RF algorithm). After establishing the relationships, the RF model was applied using the predictors to predict surface soil moisture over the globe at 1 km spatial resolution for 21 years.

## Data Records

The GSSM1 km dataset can be accessed at: https://figshare.com^45^. It contains global daily soil moisture data with a spatial resolution of 1 km, in cm^3^/cm^3^, from February 2000 to December 2020. These data are stored in GeoTiff format with one file per year and it is divided based on continents, including Europe, Africa, North America (1&2), South America, Oceania, and Asia (1&2&3&4). An example file name is “SM2002Europe1 km”, and an example of the band name is “band_2002_01_01_classification” which means soil moisture on January 1, 2002, the scale factor is 1000 (need to divide 1000). The coordinate system is WGS84 (“EPSG:4326”).

## Technical Validation

### Model testing

The performance of the RF model was tested on the testing set (64226 samples). As presented in Fig. 3, the performance has RMSE (see more details in supplementary materials section 3: Evaluation metrics) of 0.05 cm^3^/cm^3^, ubRMSE of 0.05 cm^3^/cm^3^, and r of 0.9. In addition, it is also essential to know which land surface feature has the most significant influence on SSM prediction. The feature importance ranking could also help us to understand comprehensively the underlying physics responsible for the SSM dynamics. As shown in Fig. 3, APEI is the most important explanatory variable among the 18 considered predictors, which is consistent with the physical process, followed by Evaporation (Evapo) and longitude (lon).

**Fig. 2:**
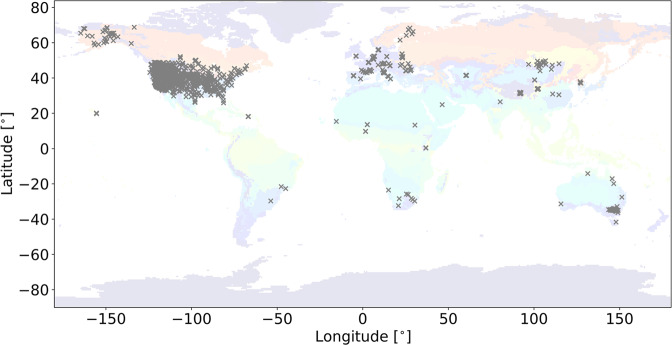
Spatial distribution of the ISMN stations.

**Fig. 3:**
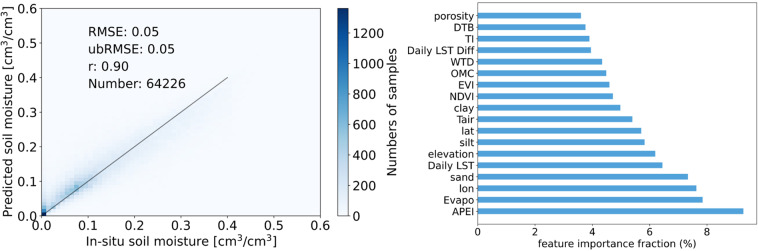
RF model testing performance and feature importance of 18 predictors.

### Time series validation

**Fig. 4:**
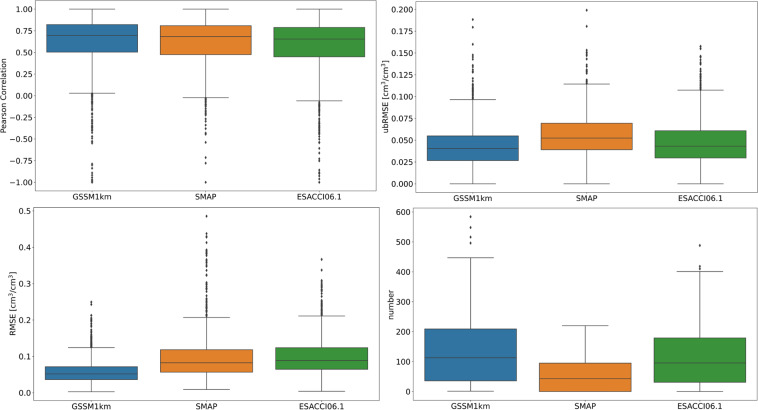
Boxplot of the metrics and number of observation of the validation set: GSSM1 km vs SMAP vs ESA-CCI06.1 at the global scale for the validation period.

**Fig. 5:**
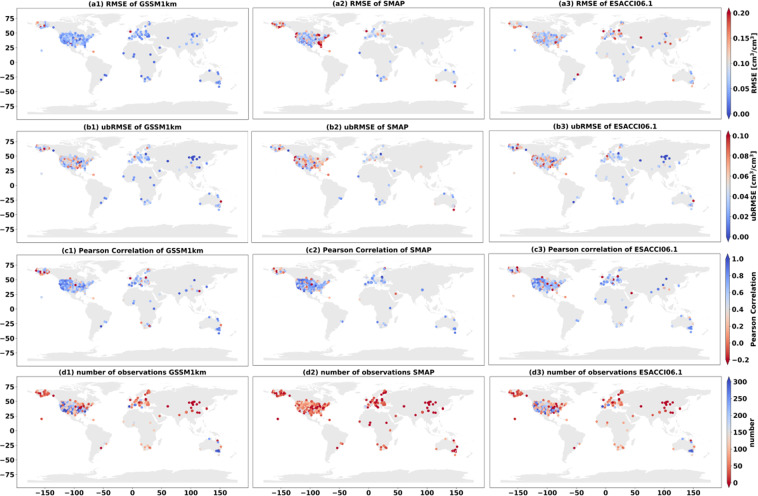
Statistical metrics distribution and number of observations in the validation set between *in-situ* SM and GSSM1 km, SMAP and ESACCI06.1 at the global scale (a1-a3: RMSE; b1-b3: ubRMSE; c1-c3: Pearson Correlation; d1-d3: number of observations).

The trained RF model was applied to the validation set. Figures 4, 5 show the boxplots and error maps of evaluation metrics among different SSM products: GSSM1 km, SMAP, and ESACCI06.1. For GSSM1 km, the median of RMSE and ubRMSE for all validation stations is 0.052 cm^3^/cm^3^ and 0.04 cm^3^/cm^3^, and the median r value for all validation stations is 0.7. SMAP shows a median of RMSE 0.082 cm^3^/cm^3^ and ubRMSE of 0.052 cm^3^/cm^3^ and a median r of 0.68 among all validation stations. ESACCI06.1 shows a median of RMSE 0.089 cm^3^/cm^3^ and ubRMSE of 0.043 cm^3^/cm^3^ and a median r of 0.65 among all validation stations. From both statistical perspective and spatial error distribution maps, GSSM1 km performs better than SMAP and ESACCI06.1.

**Fig. 6:**
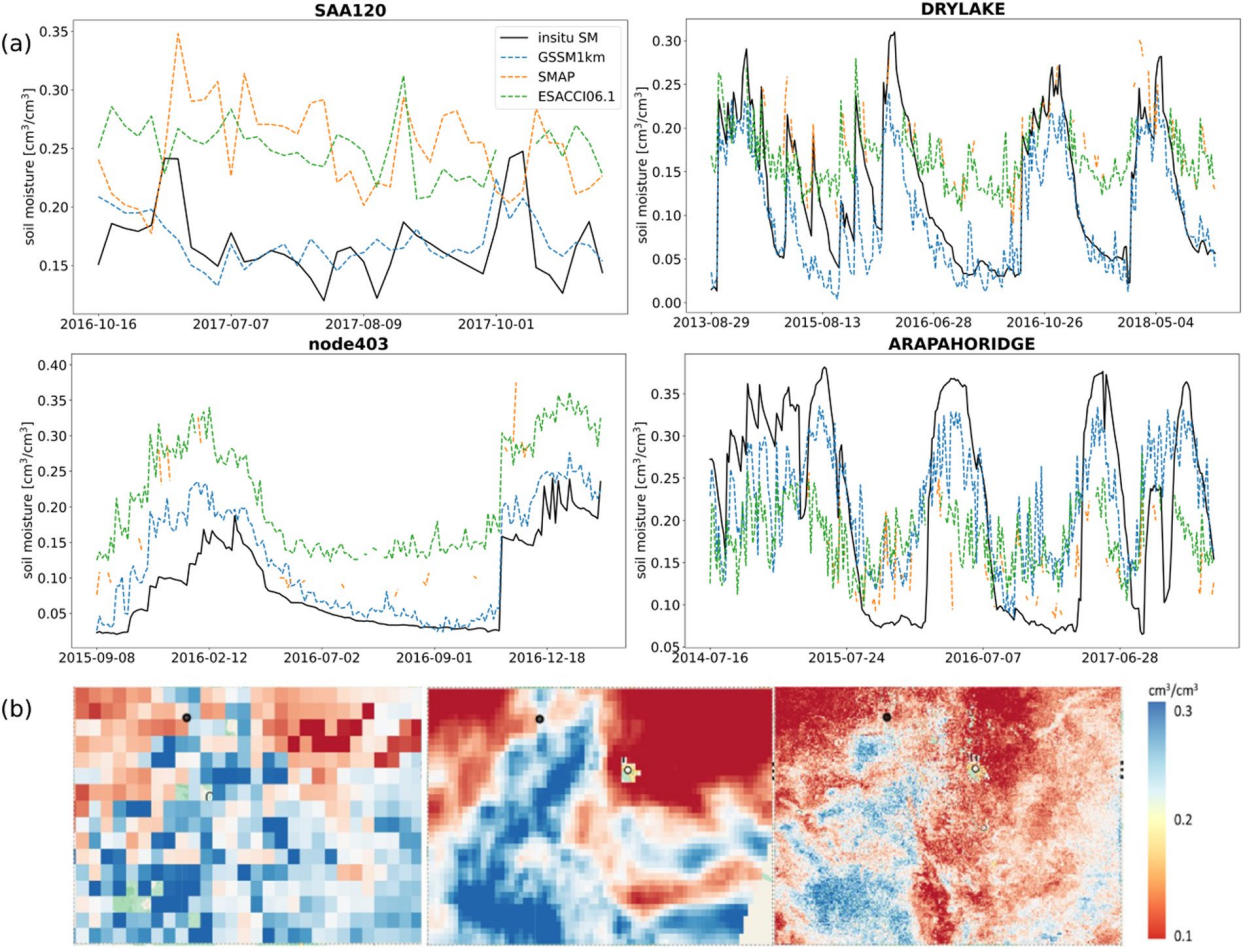
(**a**) Time series and spatial distribution in specific days of predicted SSM with ESACCI06.1, SMAP and GSSM1 km at selected stations during extreme events. (1) Station SAA120; (2) Station DRYLAKE; (3) Station node403; (4) Station ARAPAHORIDGE. (**b**) Colorado (DRYLAKE) in ESACCI06.1-0.25°, SMAP-9 km, GSSM1 km-1 km on 7 Aug, 2016.

The predicted SSM time series from GSSM1 km, SMAP, and ESACCI06.1 have been analyzed along with the *in-situ* observation to demonstrate the capability of the GSSM1 km for depicting extreme events. Figure 6a shows the comparison at four stations. In SAA120, GSSM1 km matches well with in-situ SSM, but SMAP and ESACCI06.1 overestimated SSM. In node403, GSSM1 km can capture the in-situ SSM variability while ESACCI06.1 and SMAP overestimated SSM. In ARAPAHORIDGE, GSSM1 km, SMAP and ESACCI06.1 all underestimated SSM but GSSM1 km has better performance relatively. DRYLAKE is located in Colorado, the USA. Nearly all (98 percent) of Colorado was experiencing at least abnormal dryness (D0), and 35 percent of the state was in moderate drought (D1) or severe drought (D2), most of which was occurring in the eastern half of the state in 2016^46^. There was an extreme drought on August 7, 2016 in DRYLAKE^46^. Next we present the spatial distribution of soil moisture on August 7, 2016 from ESACCI06.1, SMAP, and GSSM1 km (Fig. 6b), which demonstrates that GSSM1 km can capture extreme events and provide more spatial information than SMAP and ESACCI06.1.

### Global-scale comparison with existing gridded datasets

**Fig. 7:**
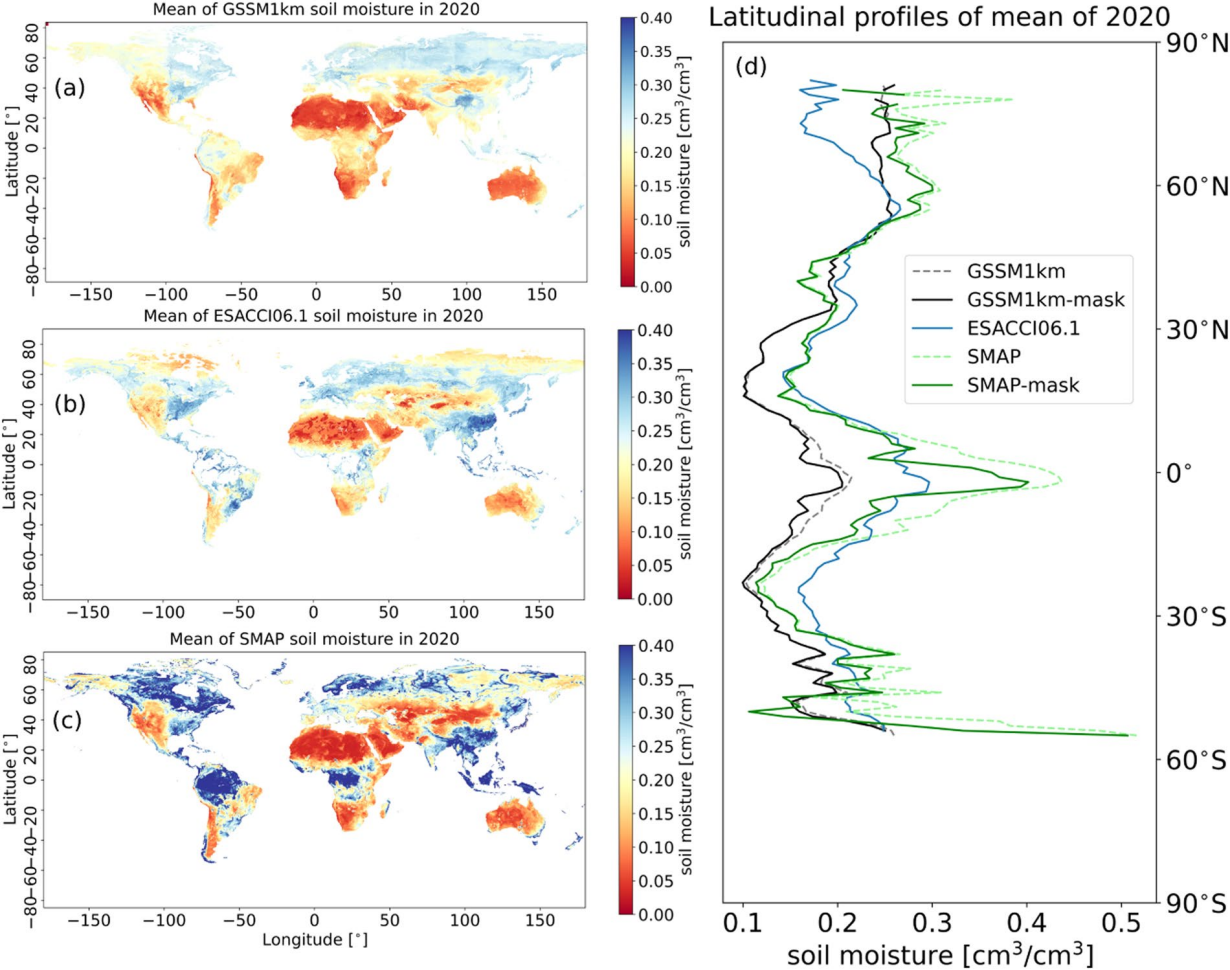
Global mean SSM map of 2020, (**a**) GSSM1 km; (**b**) ESA-CCI06.1; (**c**) SMAP. Areas in white means no data. (**d**) Comparison of latitudinal profiles among GSSM1 km, GSSM1 km-mask, ESA-CCI06.1, and SMAP, SMAP-mask. ESACCI06.1 is used as a mask for GSSM1 km and SMAP because it has missing data.

We also compared the spatial patterns of GSSM1 km with ESACCI06.1 and SMAP at the global scale. Figure 7a presents the mean soil moisture values of these three datasets in 2020 (more detail is explained in supplementary materials section 6: Latitudinal patterns, see Fig. S4). Due to the missing data of ESACCI06.1, ESACCI06.1 mean in 2020 was used as a mask to calculate the latitudinal profiles for GSSM1 km and SMAP, which is named as GSSM1 km-mask and SMAP-mask. For fair comparability, we focus our discussion on the masked result. A similar spatial pattern is observed between GSSM1 km-mask, ESACCI06.1, and SMAP-mask, but SMAP-mask is relatively wetter. For instance, the highest average soil moisture occurs near the equator in the tropics and 60° N, while the driest soil moisture is found near 20° N. Nonetheless, GSSM1 km-mask between 15° N and 15° S tends to be drier than the other two datasets. GSSM1 km-mask might be less skillful at predicting soil moisture in arid regions, due to the sparse soil moisture stations in these regions (Fig. 2). The uncertainty of GSSM1 km is described in supplementary materials section 5: Uncertainty for those regions without ground observations (Fig. S1–S3).

### Validation using stations in the Netherlands

The GSSM1 km has gone through the versions history from v1.0 to v1.7 with global training samples. Here, we focus on presenting only results from v1.2.2 to v1.7 in the Netherlands (the details of change between each version are provided in supplementary materials section 4: History of versions). The SSM networks in the Netherlands include Raam (14 stations, 2016-04-05 to 2019-04-05) and Twente (10 stations, 2016-01-01 to 2019-12-31) (referred to as NL stations). The six versions of GSSM1 km (v1.2.2 to v1.7) were produced and compared with the *in-situ* SSM in the Netherlands over the whole observation period.

**Fig. 8:**
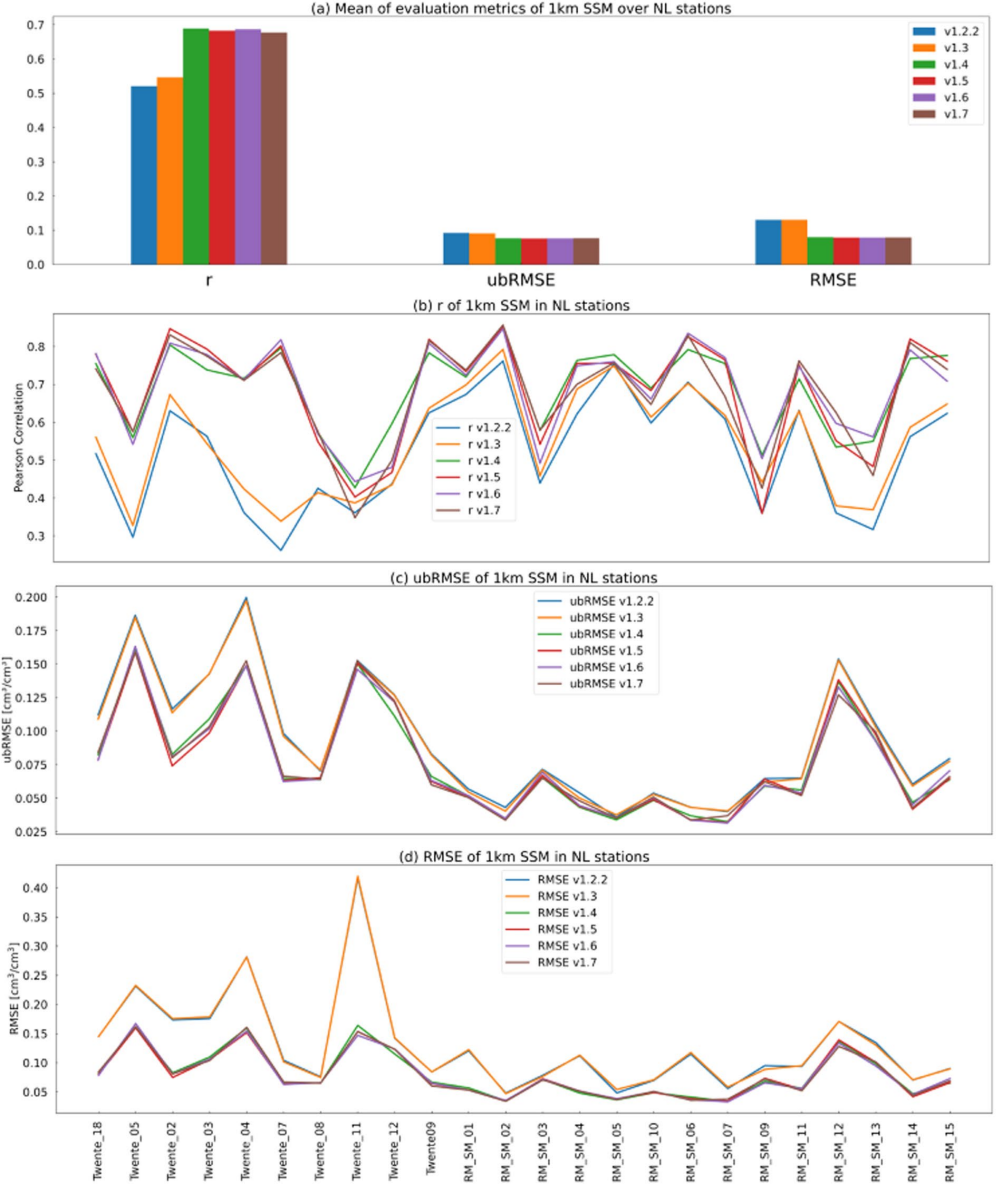
(**a**) Mean of each metric over 24 stations in each version, (**b**–**d**) Evaluation metrics of 1 km SSM (v1.2.2 to v1.7) in NL stations over the whole observation period.

**Fig. 9:**
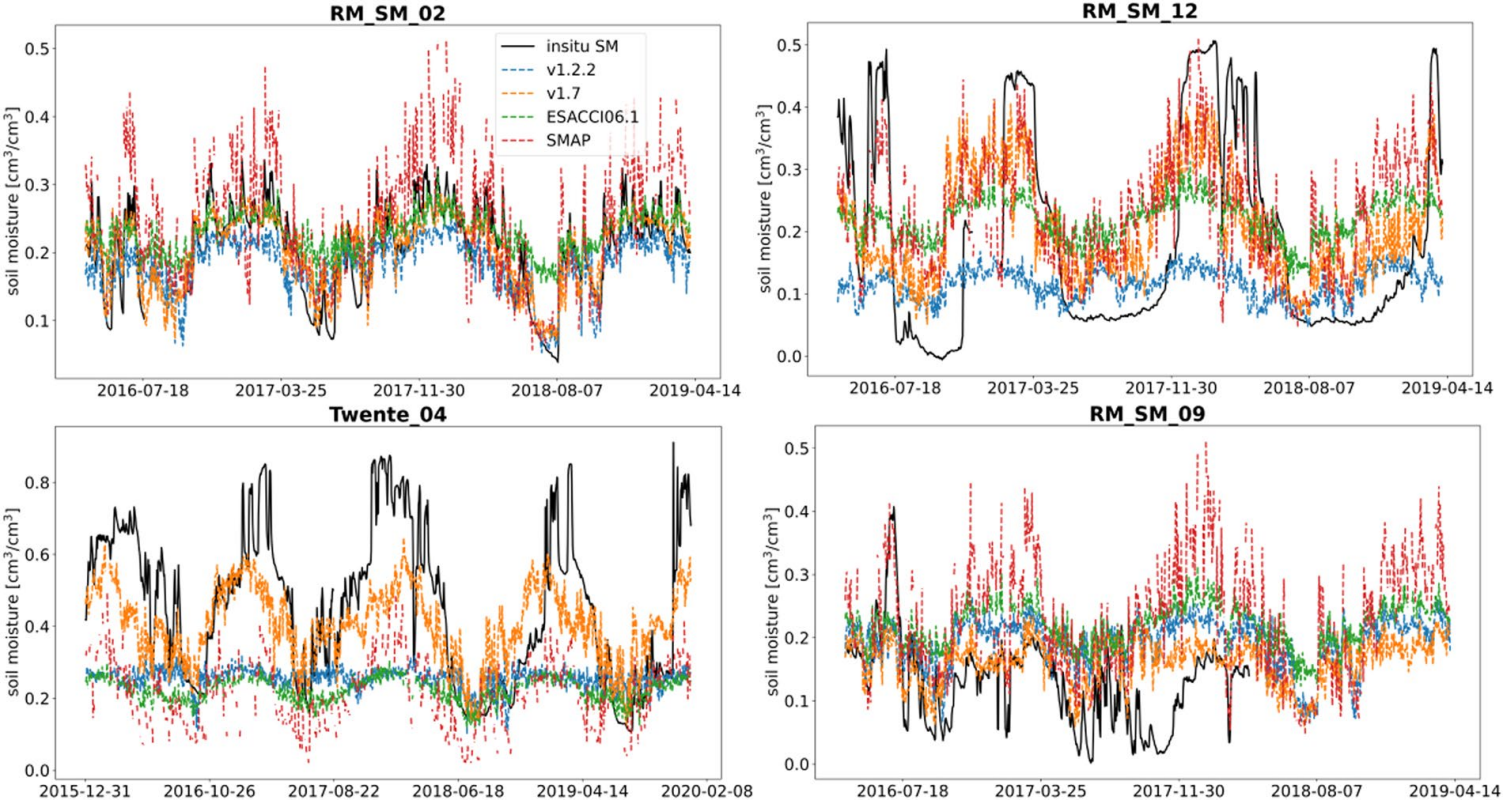
Comparison of SSM from *in-situ*, v1.2.2, v1.7, ESACCI06.1, and SMAP in NL stations over the whole observation period.

From Fig. 8a, we can see the performance was improved over the NL stations. For specific stations, some stations were improved significantly from v1.2.2 to v1.7, while others are not. RM_SM_02 was improved the most, and so did RM_SM_12 and Twente_04. RM_SM_09 is an example that did not get improved obviously (it is to note that RM_SM_12 and RM_SM_09 are at the same 25 km pixel). The soil moisture from *in-situ* SM, v1.2.2, v1.7, ESACCI06.1, and SMAP of these 4 stations are further compared (see Fig. 9 and Table 2).

**Table 2:** Evaluation metrics of v1.2.2, v1.7, ESACCI06.1, and SMAP in NL stations over the whole observation period.

		r	ubRMSE	RMSE
RM_SM_02	V1.2.2	0.76	0.04	0.05
	V1.7	**0.86**	**0.03**	**0.03**
	ESACCI06.1	0.85	0.04	0.05
	SMAP	0.79	0.06	0.07
RM_SM_12	V1.2.2	0.36	0.15	0.17
	V1.7	**0.63**	**0.13**	**0.13**
	ESACCI06.1	0.52	0.15	0.15
	SMAP	0.55	0.13	0.14
Twente_04	V1.2.2	0.36	0.2	0.28
	V1.7	0.71	**0.15**	**0.16**
	ESACCI06.1	**0.72**	0.19	0.29
	SMAP	0.7	0.16	0.29
RM_SM_09	V1.2.2	0.36	0.06	0.1
	V1.7	0.43	**0.06**	**0.07**
	ESACCI06.1	0.37	0.07	0.11
	SMAP	**0.52**	0.08	0.14

In these 4 stations, v1.7 performs better than v1.2.2 either significantly or slightly. In RM_SM_02, v1.7 is better than v1.2 and performs similarly as ESACCI06.1. In RM_SM_12, v1.7 is better than v1.2 and ESACCI06.1. In Twente_04, all datasets underestimated SSM, but relatively v1.7 has a better performance in terms of magnitude. In RM_SM_09, all datasets cannot capture the dynamic changes well but v1.7 still performs relatively better.

### Validation using stations on the tibetan plateau

**Fig. 10:**
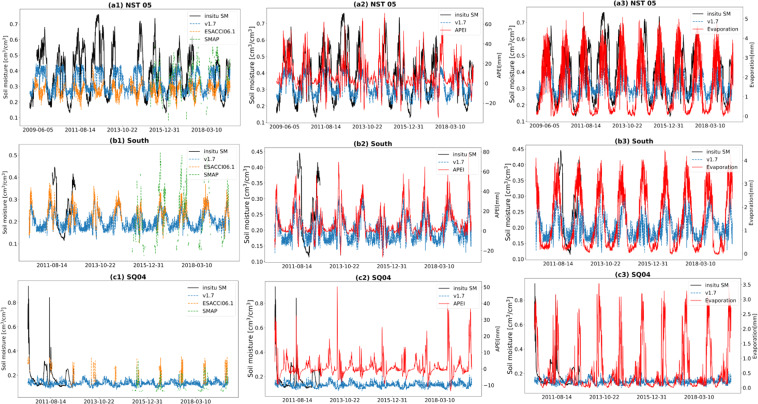
Comparison of Tibetan Plateau stations over the whole observation period. *In-situ* SSM, GSSM1 km (v1.7), ESA-CCI06.1, and SMAP (a1: NST 05, b1: South, c1: SQ04). *In-situ* SSM, GSSM1 km (v1.7) and APEI (a2: NST 05, b2: South, c2: SQ04). *In-situ* SSM, GSSM1 km (v1.7) and Evaporation (a3: NST 05, b3: South, c3: SQ04).

On the Tibetan Plateau, GSSM1 km (v1.7) was compared with the *in-situ* SM, ESACCI06.1, and SMAP over the whole observation period. There are three SM monitoring networks in Tibetan Plateau, including Maqu, Naqu, and Nagari (including Shiquanhe and Ali)^8,47^. In this study, based on the evaluation result, we choose one station from each network to do a detailed comparison: ‘NST 05’ from Maqu, ‘South’ from Naqu, and ‘SQ04’ from Nagari (Fig. 10, Table 3).

**Table 3:** Evaluation metrics of GSSM1 km (v1.7), ESACCI06.1, and SMAP at Tibetan Plateau stations over the whole observation period (Cells with a hyphen represent that no SMAP data are available).

		r	ubRMSE	RMSE
NST 05	GSSM1 km	**0.76**	0.11	0.13
	ESACCI06.1	0.4	0.11	0.24
	SMAP	0.52	**0.1**	**0.11**
South	GSSM1 km	**0.59**	0.09	**0.11**
	ESACCI06.1	0.4	**0.05**	0.13
	SMAP	—	—	—
SQ04	GSSM1 km	−0.03	**0.11**	**0.12**
	ESACCI06.1	**0.65**	0.18	0.2
	SMAP	—	—	—

At station NST 05 (Fig. 10a1), GSSM1 km and ESACCI06.1 both underestimated SSM but GSSM1 km performs better. SMAP lacks data in most days, but in the days it has data, it performs similarly to GSSM1 km. At station South (Fig. 10b1), SMAP does not have data when there is *in-situ* SSM. GSSM1 km is better at capturing dynamics, but ESACCI06.1 is better at the magnitude. At station SQ04 (Fig. 10c1), SMAP does not have data when there is *in-situ* SSM. ESACCI06.1 can capture 3 peaks (less than 0.4 cm^3^/cm^3^) of high SSM, which leads to a better consistency metric. However, both GSSM1 km and ESACCI06.1 cannot capture the SSM higher than 0.4 cm^3^/cm^3^. The possible reason we found is the APEI (Fig. 10c2) in this station is relatively lower than the APEI in South and NST and APEI has a positive relationship with SSM.

## Usage Notes

We present a global, long term, daily 1 km surface soil moisture dataset generated through a physics-informed ML algorithm, constrained with *in-situ* measurements. Our GSSM1 km dataset outperforms other existing gridded datasets, in terms of daily temporal dynamics as shown by the highest temporal correlation with the *in-situ* measurements. Nevertheless, under conditions for those regions outside the spatiotemporal range sampled by the *in-situ* measurements, the uncertainties of the GSSM1 km are difficult to be determined.

RF performance can be significantly affected by the lack of diversity in the training data. As shown in Fig. 3, although the *in-situ* soil moisture measurements were obtained from global networks, the data did not cover all climate zones across the globe. Therefore, outside of the training conditions such as high latitudes and in arid regions, relatively high uncertainty is expected. The lack of observations under specific conditions poses the same challenges for other datasets and models. Therefore, using GSSM1 km in an ensemble of differently derived datasets may help obtain more reliable soil moisture information in these data-sparse regions. The new soil moisture dataset is an important complement to the existing suite of soil moisture datasets and can enhance the future large-scale analysis of extreme events.

The data source (satellite, reanalysis data, and other data), data processing (including pre-processing of predictors, spatial resampling of predictors, and samples splitting), evaluation metrics, history of versions, uncertainty, and latitudinal patterns are given in the supplementary materials.

## Supplementary information

Supplementary materials

## Data Availability

All the codes used in this study to generate the dataset were written in the Javascript in Google Earth Engine and are available through GitHub (https://github.com/AliciaPython/GSSM1km). The GSSM1 km dataset can be accessed at: https://code.earthengine.google.com/?asset=users/qianrswaterr/GlobalSSM1km0509.
