# Supplementary material for: Global long term daily 1 km surface soil moisture dataset with physics informed machine learning
Source: Sci Data. 2023 Feb 17;10:101. doi: 10.1038/s41597-023-02011-7 (PMC9938112; doi:10.1038/s41597-023-02011-7)
Supplement: Supplementary file 1 — Supplementary materials [file 41597_2023_2011_MOESM1_ESM.docx]

**Global long term daily 1km surface soil moisture dataset with physics informed machine learning**

Qianqian Han^1^, Yijian Zeng^1^, Lijie Zhang^2^, Chao Wang^3^, Egor Prikaziuk^1^, Zhenguo Niu^4^, Bob Su^1,5,*^

1. Faculty of Geo-Information Science and Earth Observation (ITC), University of Twente, 7514 AE Enschede, The Netherlands

2. Research Center Jülich, Institute of Bio- and Geosciences: Agrosphere (IBG-3), 52428 Jülich, Germany

3. Department of Earth, Marine and Environmental Sciences, University of North Carolina, Chapel Hill, NC, USA

4. State Key Laboratory of Remote Sensing Science, Aerospace Information Research Institute, Chinese Academy of Sciences, Beijing 100101, China

5. Key Laboratory of Subsurface Hydrology and Ecological Effect in Arid Region of Ministry of Education, School of Water and Environment, Chang’an University, Xi’an 710054, China

Corresponding author: Bob Su ([z.su@utwente.nl](mailto:z.su@utwente.nl))

Contents

[1 Satellite and reanalysis data 2](#_Toc126239802)

[2 Data processing 3](#_Toc126239803)

[2.1 Data pre-processing 3](#_Toc126239804)

[2.2 Spatial resampling 5](#_Toc126239805)

[2.3 Data splitting 5](#_Toc126239806)

[3 Evaluation metrics 5](#_Toc126239807)

[4 History of versions 6](#_Toc126239808)

[5 Uncertainty for those regions without ground observations 6](#_Toc126239809)

[6 The latitudinal patterns 13](#_Toc126239810)

[References 13](#_Toc126239811)

**Supplementary materials**

# 1 Satellite and reanalysis data

1. Satellite data

The input MOD11A1.006 Terra Land Surface Temperature and Emissivity Daily Global 1km is a product produced by NASA. It consists of day and night LST, and emissivity. The time period of this dataset is from February 2000 to present. The temporal resolution of this dataset is daily and the spatial resolution is 1 km. In our study, the bands used from this dataset are day and night LST, which are major variables useful in the studies of climate, hydrology, greenhouse effect, and many other related fields ^1^. The MOD11A1.006 dataset presented in this study is available at <https://developers.google.com/earth-engine/datasets/catalog/MODIS_006_MOD11A1?hl=en>.

The input MOD13A2.006 Terra Vegetation Indices 16-Day Global 1km is another product produced by NASA for analysing the change in vegetation with two indices: Enhanced Vegetation Index (EVI) and Normalized Difference Vegetation Index (NDVI). The indices are available from February 2000 to present and used for depicting spatial and temporal variations in vegetation with sixteen days interval and 1 km spatial resolution. The MOD13A2.006 dataset presented in this study is available at <https://developers.google.com/earth-engine/datasets/catalog/MODIS_006_MOD13A2?hl=en>.

1. Reanalysis data

The input European ReAnalysis Land (ERA5Land) describes the evolution of water and energy cycles over land in a consistent manner over the production period, which, among others, could be used to analyse trends and anomalies ^2^. The spatial and temporal resolution used in this study is 11km and hourly. It has 69 variables available from 1981 until now. Total_precipitation, total_evaporation, temperature_2m were used in this study. The ERA5Land dataset presented in this study is available at <https://developers.google.com/earth-engine/datasets/catalog/ECMWF_ERA5_LAND_HOURLY?hl=en>.

1. Hydrogeological data

Water Table Depth (WTD) is a global static dataset with 1 km spatial resolution ^3^. This dataset was generated based on the compiled global observations of WTD from government archives and literature using a groundwater model forced by modern climate, terrain, and sea level. The patterns in WTD can help explain patterns in wetlands at the global scale and vegetation gradients at regional and local scales ^3^. The WTD is available at <https://aquaknow.jrc.ec.europa.eu/en/content/global-patterns-groundwater-table-depth-wtd>.

Depth to Bedrock (DTB) is a global static dataset with 3 level spatial resolution (250 m, 1 km, 10 km) and it has 3 bands: absolute DTB, censored DTB, occurrence of R horizon ^4^. This dataset was generated by Random Forest (RF) and Gradient Boosting Tree Algorithms. Absolute DTB with 1 km resolution was used in this study. The DTB is available at <http://globalchange.bnu.edu.cn/research/dtb.jsp>.

1. Terrain and soil data

MERIT Hydro: Global Hydrography Datasets is a new global flow direction map at 90 m resolution at the equator derived from the version 1.0.3 of the MERIT DEM elevation data and water body datasets (G1WBM, GSWO and OpenStreetMap). Elevation, flow direction, upstream drainage area (flow accumulation area) were used in this study. The MERIT Hydro is available at <https://developers.google.com/earth-engine/datasets/catalog/MERIT_Hydro_v1_0_1?hl=en>.

SoilGrids provides global predictions for standard numeric soil properties (organic carbon, bulk density, Cation Exchange Capacity, pH, soil texture fractions and coarse fragments) at 250 m resolution and seven standard depths (0, 5, 15, 30, 60, 100 and 200 cm) ^5^. In this study, organic carbon, bulk density, soil texture fractions were used.

(5) Other SM products for comparison

Two SM products were used for the spatio-temporal comparison with GSSM1.7. European Space Agency Climate Change Initiative (ESA CCI) soil moisture product combines various single-sensor active and passive microwave soil moisture products into three harmonised products: a merged ACTIVE, a merged PASSIVE, and a COMBINED active+passive microwave product ^6^. This study used SM data of ESA CCI combined version 06.1, with the temporal coverage from January 2000 to December 2020. This dataset is available at <https://www.esa-soilmoisture-cci.org/v06.1_release>.

Soil Moisture Active Passive (SMAP) mission launched in January 2015 delivers data products at 4 levels ^7^. It carries an L-band radiometer and an L-band high-resolution radar to estimate near-surface soil moisture. In this study, SMAP Enhanced L3 Radiometer Global and Polar Grid Daily 9 km EASE-Grid Soil Moisture V005 was selected for comparison. The dataset is available at <https://nsidc.org/data/SPL3SMP_E/versions/5>.

# 2 Data processing

## 2.1 Data pre-processing

1) APEI

The APEI was calculated from ERA5Land precipitation and evaporation. The definition of APEI at day *t* can be represented as equation (1) below:

$APEI_{t}=\sum_{i=0}^{N} k^{i}\cdot{{(p}_{t-i}-e}_{t-i})$ (1)

Where k is an empirical factor to indicate the decay effect from the rainfall, which should always be less than one, a suggested range of the decay parameter is between 0.85 and 0.98 ^8^, where APEI_t_ is the APEI value at day of t, and p_t-i_, e_t-i_ is the precipitation and evaporation value respectively at i^th^ days before the day of t. N is the maximum number of days before the day of t.

Although spatial heterogeneity exists in the parameters of the APEI equation, it is not possible to determine the parameters for every single location since the retention of water in the soil is heterogeneous from space, and most studies only use one pair of values to represent a specific region^9^. In this study, we also determined only one pair of optimized parameters for the entire study area. Here, we calculated the APEI with different combinations of the parameters and compared the APEI with the in-situ SM utilizing the Pearson Correlation Coefficient (r), and we chose the optimized parameters when r reaches the highest value. However, only the data from the network USCRN were used, which is one of the most important long-term in-situ soil moisture measurement networks dedicated for climate studies. The optimized value for k is 0.91, and for N is 33, we considered cumulative effects of precipitation and evaporation in 34 days (0-33).

2) Evaporation, Tair

Evaporation and Tair are from ERA5Land, with hourly temporal resolution. Evaporation in ERA5Land is the accumulated amount of water that has evaporated from the Earth's surface, including a simplified representation of transpiration (from vegetation), into the air above. Tair is temperature of air at 2m above the surface of land, sea or in-land waters. We composited them from hourly into daily, cumulative for evaporation and average for Tair.

3) Daily LST, Daily LST Difference

The MOD11A1 Level 3 LST product of version 6 from the Terra polar-orbiting NASA sun-synchronous satellite (10:30 AM/PM local time) was used in this study. Provided with the LST from both daytime and night-time, the associated quality control (QC) assessments was used to ensure the quality of the LST in the samples. Only the pixels with the QC_Day and QC_Night value of 0 (i.e., good quality data) were kept. The original unit is Kelvin with a scale factor 0.02, which was converted to Celsius by multiplying 0.02 and subtracting 273.15. We considered the arithmetic average of day LST and night LST as daily LST and calculated the difference between the daytime and night-time as the Daily LST diff for that day.

However, there are a lot of gaps existing in MOD11A1 because of clouds. It is necessary to fill in these gaps if we want to get a spatially continuous 1km SM product. An algorithm was proposed to fill these gaps in MYD11A1 based on Temporal Fourier Analysis, with the help of air temperature data CFSV2 (Climate Forecast System Version 2) ^10^ (see equation 2), for the pixels where MOD11A1 has data, we kept the original LST from MOD11A1.

${LST}_{cont}\left( t \right)={LST}_{clim}\left( t \right)+T_{anom}(t)$ (2)

Where LST_cont_(t) is the actual LST at time t, LST_clim_(t) is the MODIS climatological LST from Temporal Fourier Analysis (TFA) , T_anom_(t) is the CSFv2 temperature anomaly which is calculated by subtracting T_clim_ (CFSv2 climatological values) from CFSv2 temperature.

4) NDVI, EVI

Both NDVI and EVI are from MOD13A2 and MODIS 16-days’ composite data. Despite an atmospheric correction procedure from the MODIS reflectance data, noise could still be observed in the long-term time series, which is considered inconsistent with plant phenology. Thus, the Savitzky-Golay (S-G) filtering method was applied to reduce the small peak noise through a smoothing procedure ^11^. Then we interpolated NDVI and EVI to a daily temporal resolution using a simple linear interpolation to synchronize the temporal resolution with other predictors as follows.

$p\left( t \right)=f\left( t0 \right)+(f\left( t1 \right)-f(t0)(\frac{t-t0}{t1-t0}))$ (3)

Where p(t) is the interpolated value, f(t0) and f(t1) are the value at time t0 and t1 respectively.

5) TI

The TI ^12,13^ of TOPMODEL is defined as ^14^:

$TI=\ln\left( \frac{\alpha}{tan\beta} \right)$ (4)

where α is local upslope catchment area per unit contour length and β is slope angle of ground surface, and can be obtained from elevation data.

$\alpha= \frac{uca}{fw}$ (5)

$fw=\left\{ \begin{aligned} 90m, when flow direction is 1 or 4 or 16 or 64, \\ \sqrt{90} m,when flow direction is 2 or 8 or 32 or 128 \end{aligned} \right.$ (6)

where uca is upslope catchment area, fw is flow width.

Uca, flow direction, elevation can be found and used directly in MERIT Hydro: global hydrography datasets ^15,16^.

6) OMC, porosity

Porosity can be inversely related to soil dry bulk density and was calculated from bulk density ^17,18^.

$\emptyset=1-\frac{\rho_{b}}{\rho_{s}}$ (7)

Where $\rho_{b}$ is the dry bulk density ($g {cm}^{-3}$) and $\rho_{s}$ is the mineral particle bulk density valued at 2.65 $g {cm}^{-3}$. For soil mixture, the bulk density scheme assumed that the coarse and fine components share the same particle density.

The OMC is related to the organic carbon content (OCC). It was calculated by multiplying OCC with the commonly used conversion factor of 1.72, which assumes that organic matter has 58% organic carbon ^19^.

## 2.2 Spatial resampling

Predictor variables have different spatial resolutions. The variables were all resampled into 1 km firstly. Then 18 variables (except longitude and latitude) were extracted for pixels at 1km that collocate with the in situ stations for RF model training and validation. The World Geodetic System of 1984 (EPSG: 4326) was chosen as the geographic coordinate system in our study.

## 2.3 Data splitting

All 18 predictors were synchronized based on the temporal coverage of in situ data time-series of each ISMN station. The strategy of data split was: First, divide the predictors and SSM into training & testing set (70%) and validation set (30%) based on the time series. For example, assuming the data were recorded from 1 January 2000 to 31 December 2019, the training & testing set consists of the first 70% data (14 years, from 2000 to 2013), and the validation set consists of the last 30% data (6 years, from 2014 to 2019). Second, split the training & testing set into two parts (e.g., training set and testing set) randomly with the proportion of 75% and 25% (in RF algorithm).

The total stations and samples for training & testing are 1725 and 514589. Due to the computation limit, 50% samples were selected to train and test, the stations and samples used are 1704 and 257811. The training and testing were split by 75% and 25%, and the numbers are respectively 193585 and 64226. The validation samples set is 1676 stations and 218735 samples.

Table 1 The number of stations and samples

|  | Training & testing  (stations, samples) | Validation  (stations, numbers) |
| --- | --- | --- |
| Global | 1725, 514589 | 1676, 218735 |
| Used in global | 1704, 257811 (193585+64226) | 1676, 218735 |

# 3 Evaluation metrics

To assess the performances of Random Forest, we compared predicted SSM against in-situ SSM. In this research, three commonly used statistical evaluation metrics ^20^ were employed: Root Mean Square Error (RMSE), unbiased Root Mean Square Error (ubRMSE), Pearson Correlation coefficient (r), where the Mean Difference (MD) is used to calculate the ubRMSE, as follows.

$RMSE=\sqrt{\frac{\sum_{i=1}^{N} (y_{pred,i}-y_{ref,i})^{2}}{N}}$ (8)

$r=\frac{\sum_{i=1}^{N} (y_{pred}-\overline{y_{pred}})(y_{ref}-\overline{y_{ref}})}{\sqrt{\sum_{i=1}^{N} (y_{pred}-\overline{y_{pred}})^{2}}\sqrt{\sum_{i=1}^{N} (y_{ref}-\overline{y_{ref}})^{2}}}$ (9)

 (10)

$ubRMSE=\sqrt{(RMSE)^{2}-(MD)^{2}}$ (11)

Where$y_{pred,i}$ is the predicted SSM, $y_{ref,i}$ is the reference in-situ SSM, N is the number of valid pairs of SSM data, $\overline{y_{pred}}$ is the mean value of the predicted SSM data.

# 4 History of versions

The history of versions is as follows. In v1.2.2, 1 km gridded API (Antecedent Precipitation Index) and samples’ API (at station scale) were calculated using ERA5-Land precipitation data. In v1.3, 1 km gridded API was calculated using KNMI for the Netherlands. It is to note that API for ISMN stations was not changed, and was still calculated with ERA5-Land data. In v1.4, in-situ soil moisture data in the Netherlands was added into the training and testing. In this version, the 1km gridded API was calculated by KNMI for the Netherlands. However, the API in ISMN stations (including NL stations) was not changed, and was still calculated by ERA5Land.

In v1.5, API at ISMN stations (including NL stations) and gridded API at 1km scale were both replaced by APEI, which is calculated using ERA5-Land precipitation and actual evaporation data. The unit of evaporation was changed from *m* into *mm*, and it was multiplied -1 to become positive, because negative values was used in ERA5-Land for evaporation. In v1.6, water table depth was added into the training. In v1.7, Depth to bedrock was added into the training.

# 5 Uncertainty for those regions without ground observations

There are 29 climate zones in total in Koeppen Geiger classification system. Our ground observations cover 19 of them (Fig. S1).


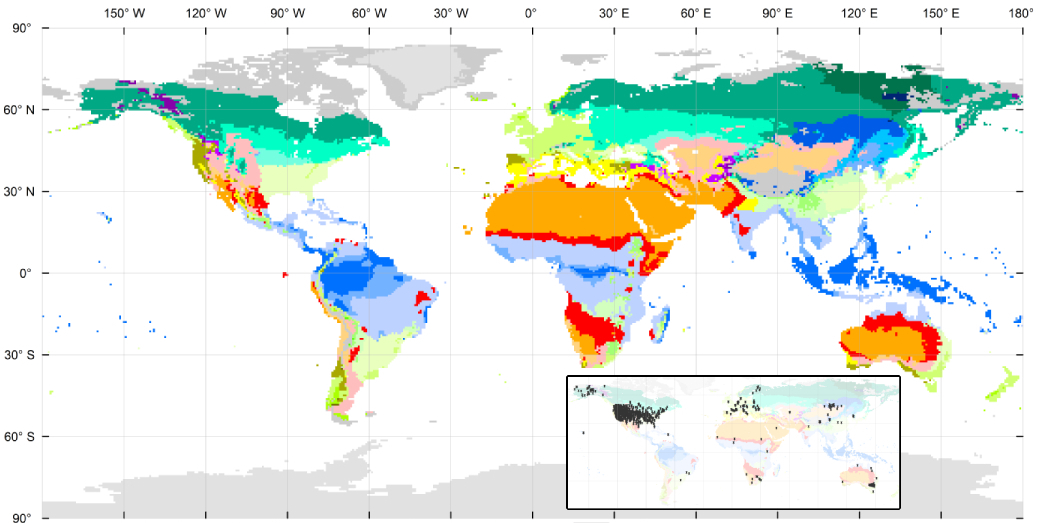

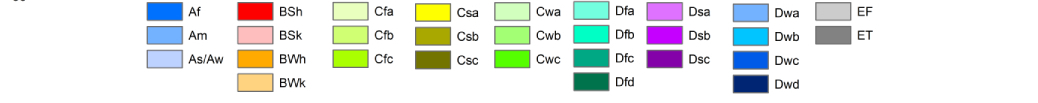


Fig. S1 KoeppenGeiger climate zones classification (Subplot in lower right corner: Spatial distribution of the ISMN stations over various climate zones)

We used Kernel density estimation to plot the probability density function (PDF) of in-situ SM and GSSM1km, both in all in-situ SM and validation set. Then we calculated the PDF overlap of in-situ SM and GSSM1km in all in-situ SM and validation set (Fig. S2-S3). For those regions without ground observations in these 19 climate zones, the PDF of in-situ SM and GSSM1km are overlapped well across all the in-situ SM (includes training, testing, and validation) and for the validation set in-situ SM (includes only validation set which were not used for training). The PDF overlap of all in-situ SM and GSSM1km average across 19 climate zones is 84%. For the validation set in-situ SM, the overlap is 80%. This means our GSSM1km can capture SM variation well in these 19 climate zones. Except in Cwb and Dwb, the overlap on the validation set is low, because there are only 3 ISMN stations in Cwb and Dwb, respectively. This means our GSSM1km has higher uncertainty in these two climate zones.


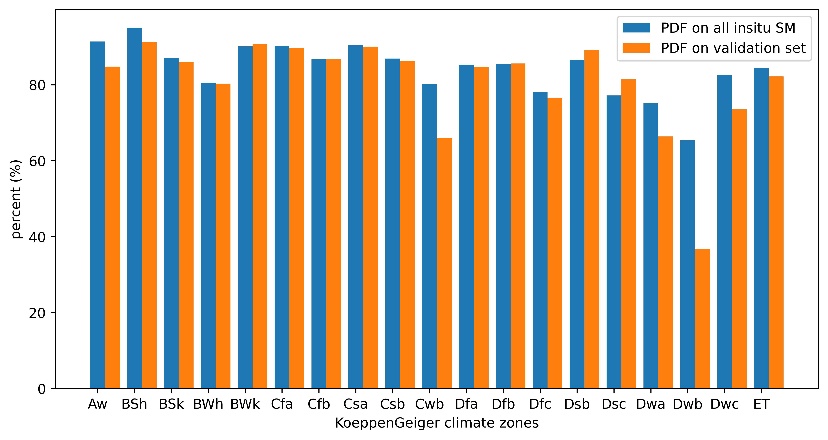


Fig. S2 Overlap of PDFs of in-situ SM and GSSM1km (blue: on all in-situ SM, orange: on validation set)


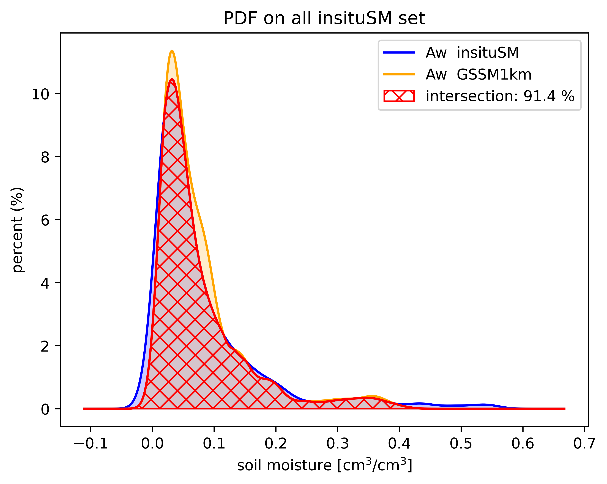

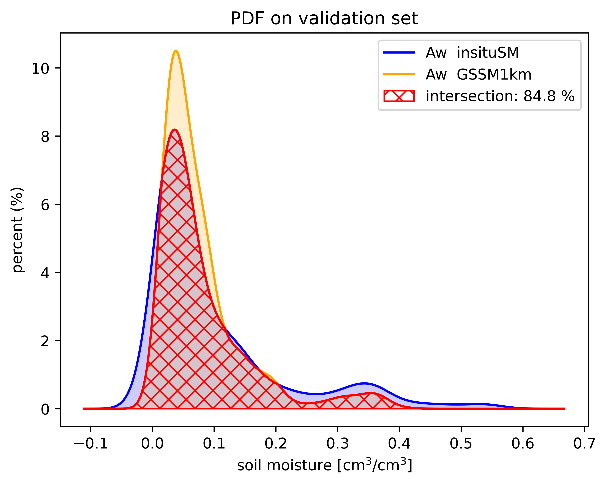

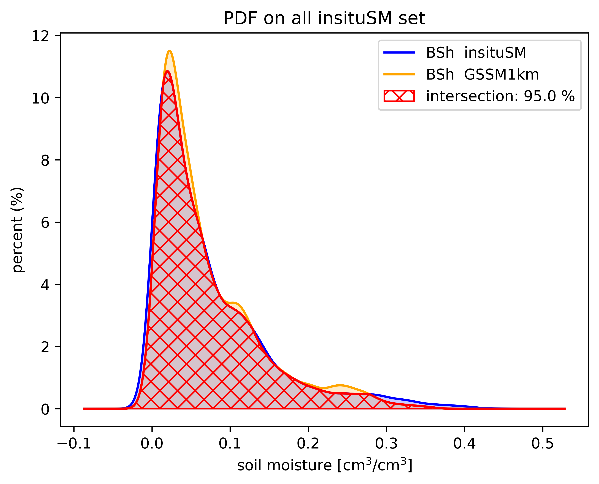

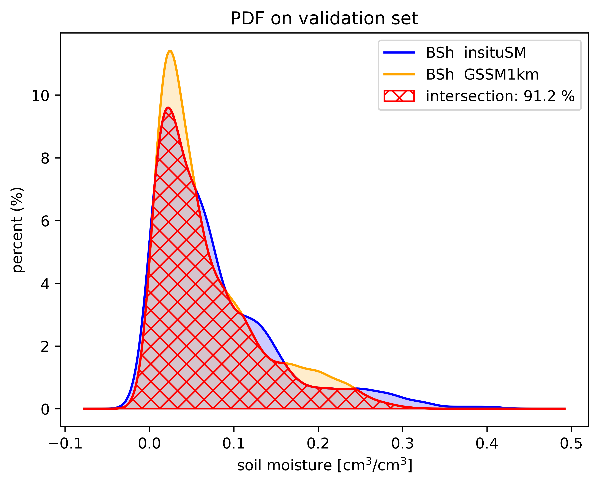


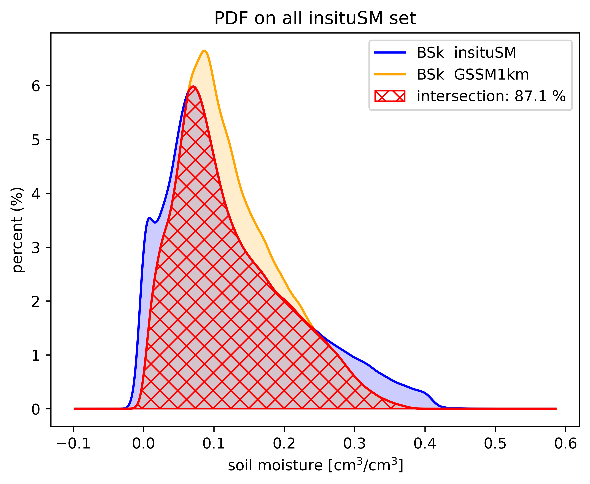

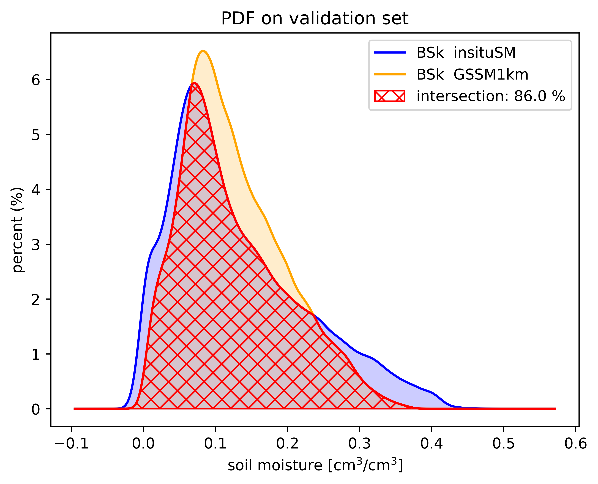

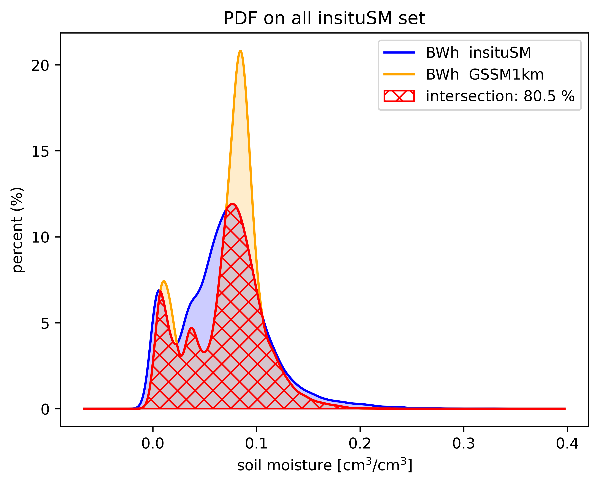

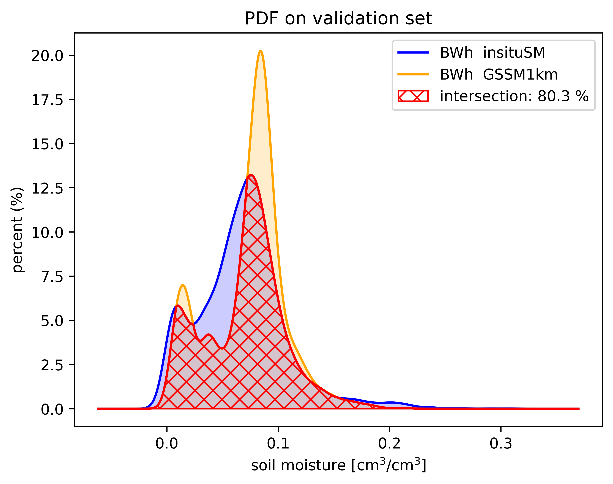

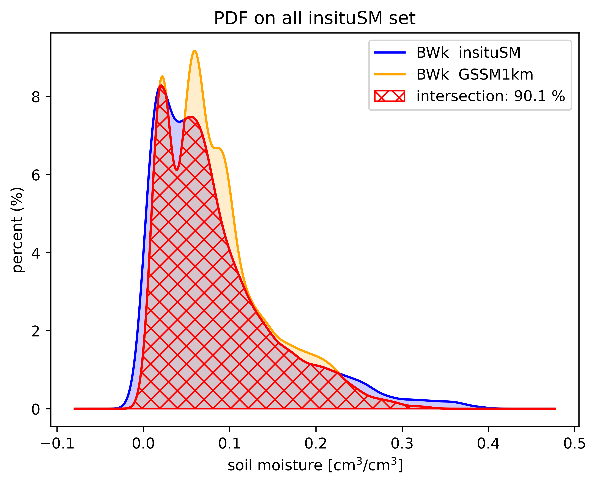

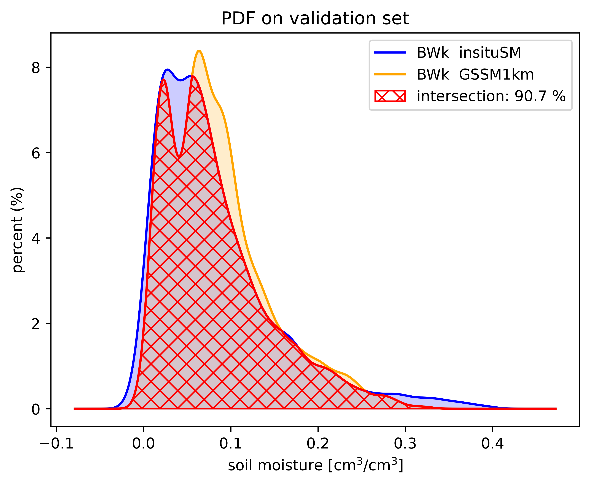

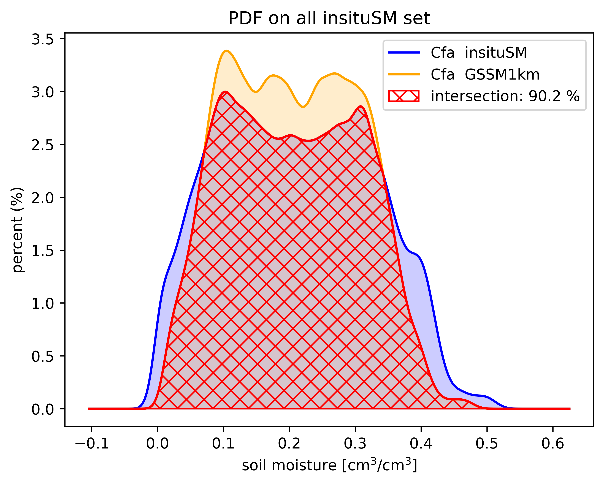

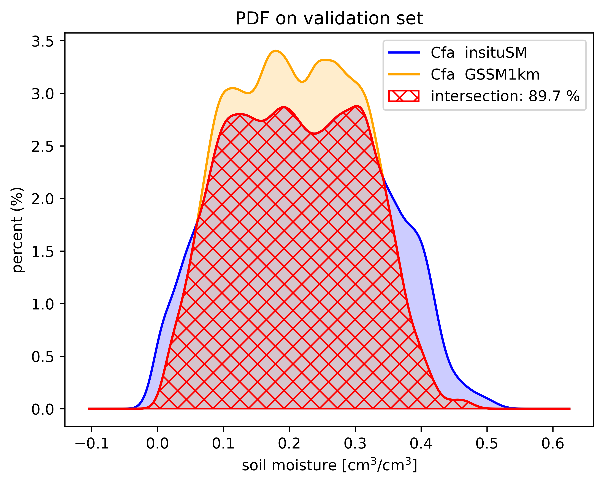

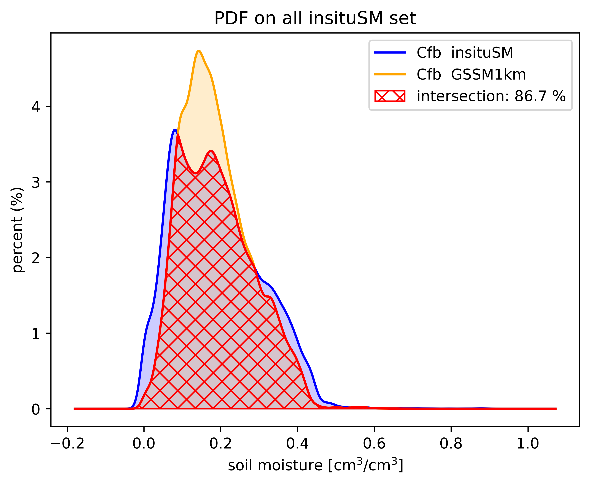

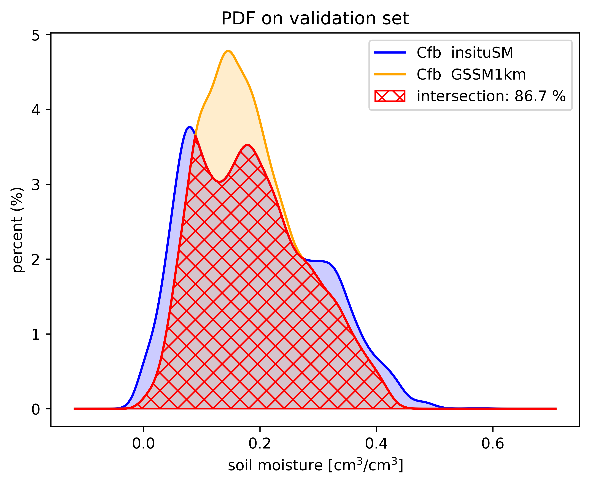

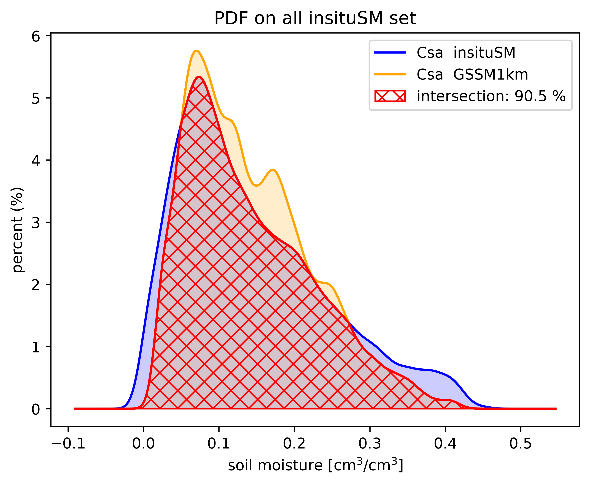

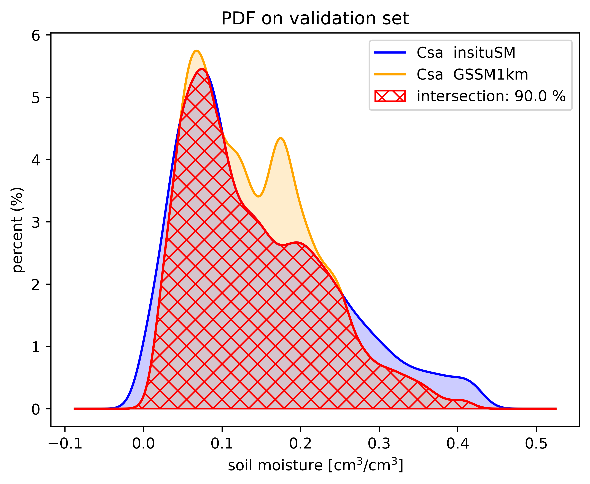

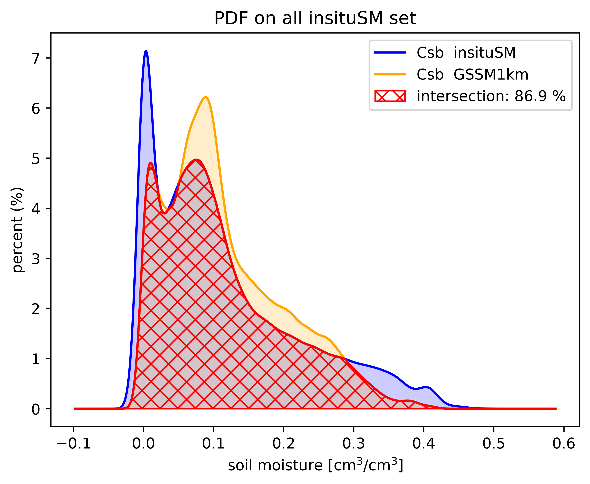

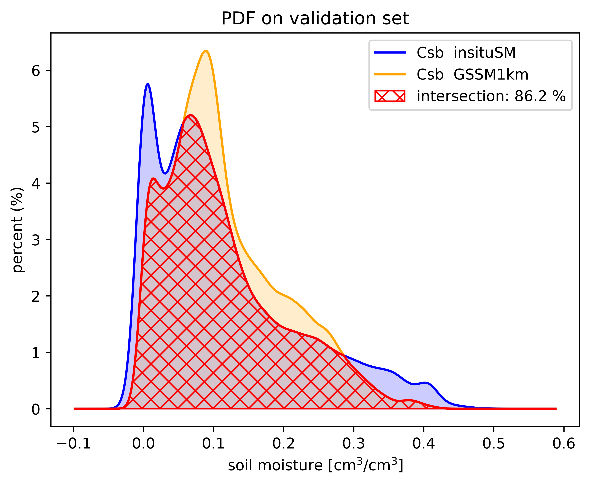

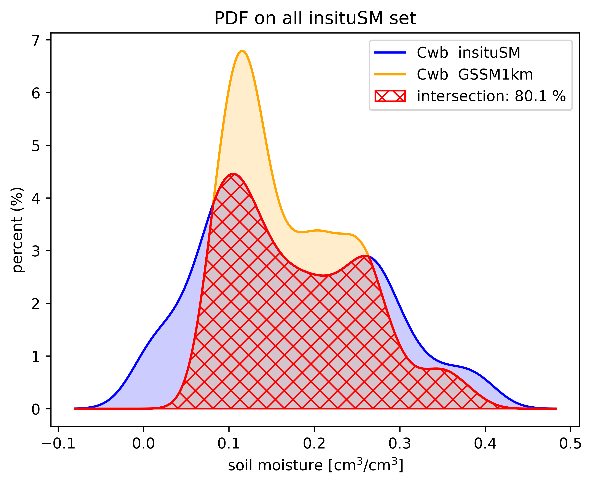

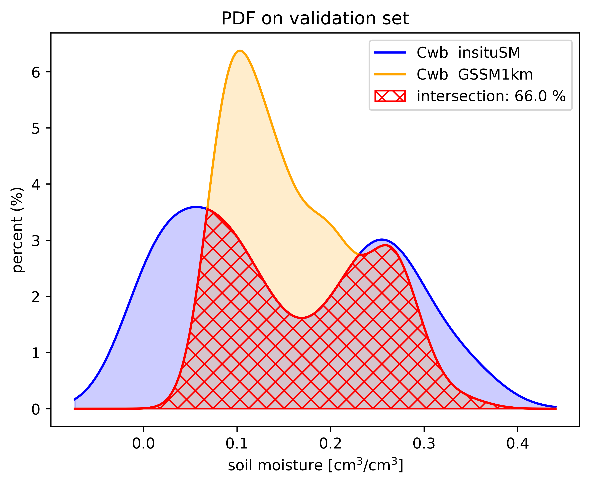

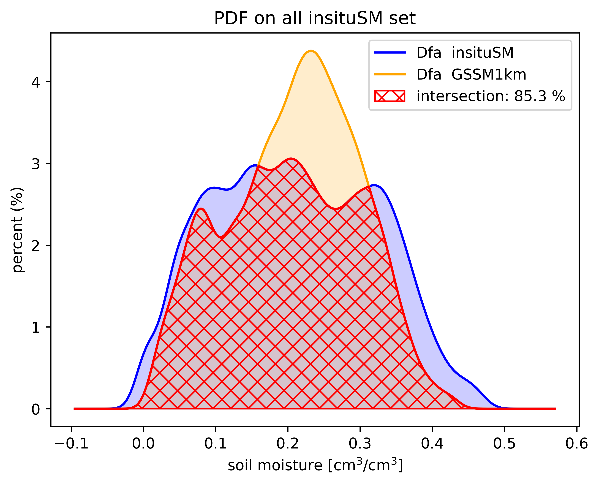

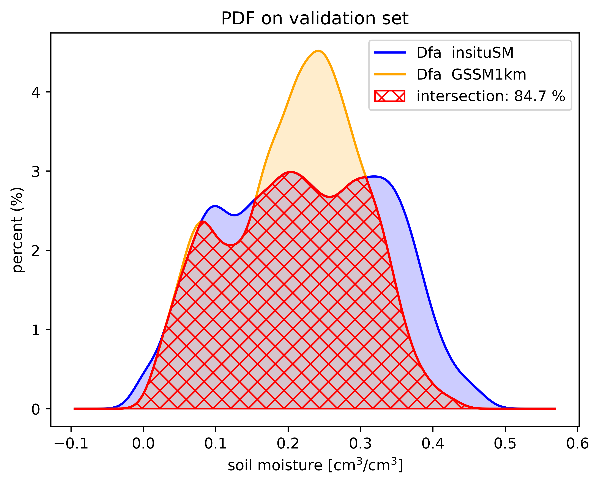

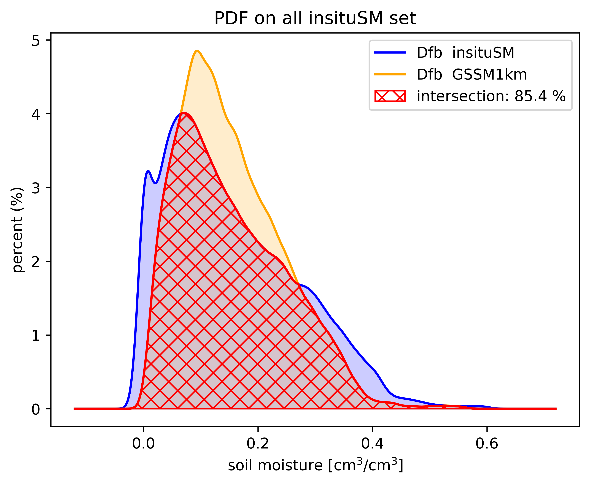

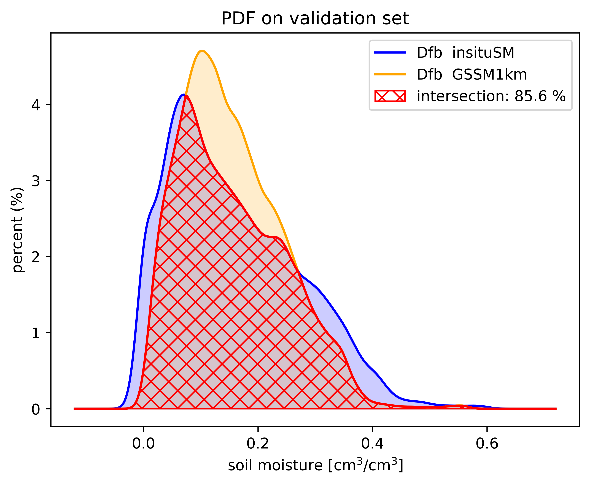

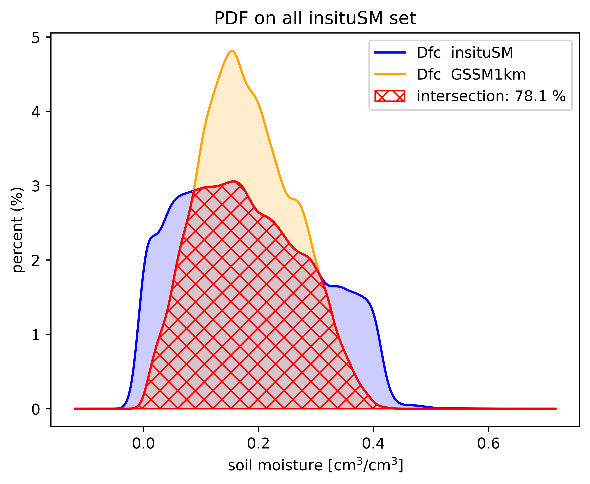

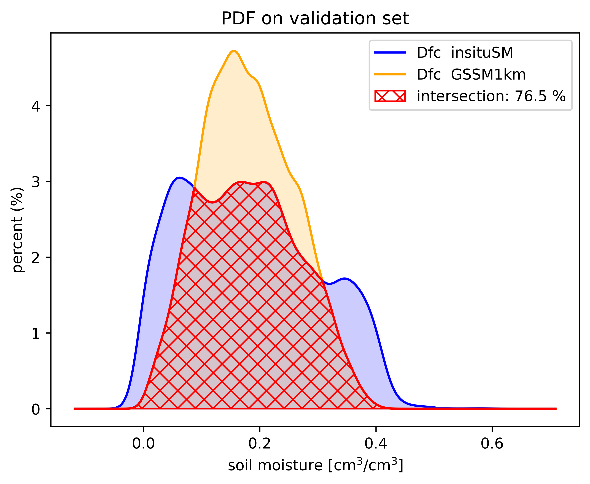

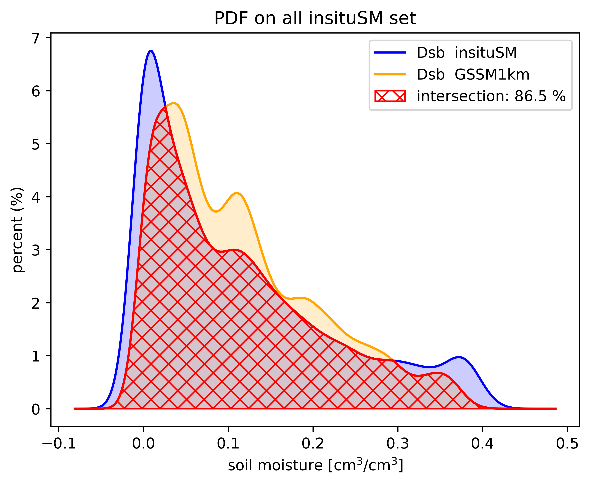

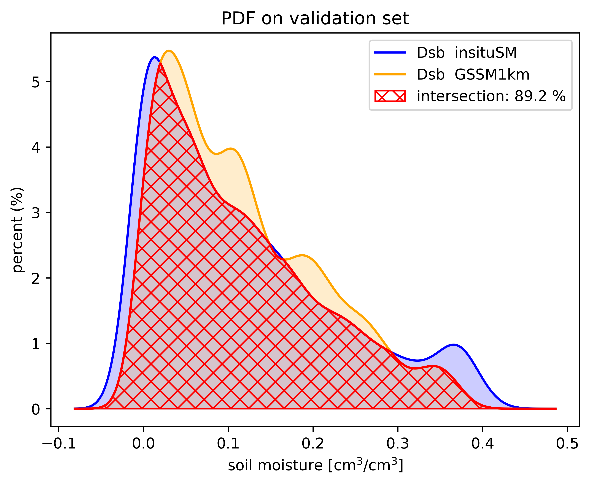

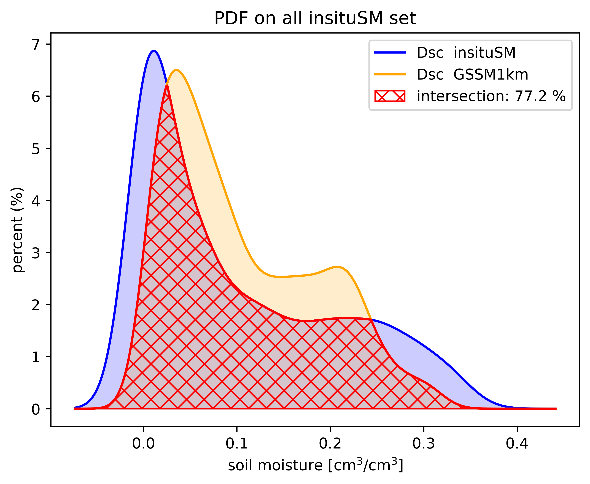

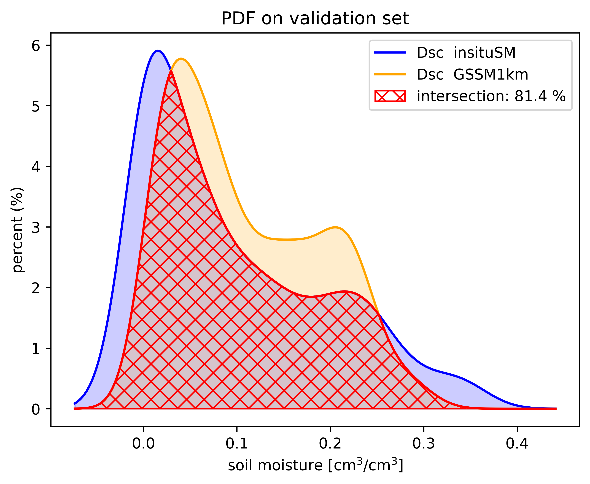

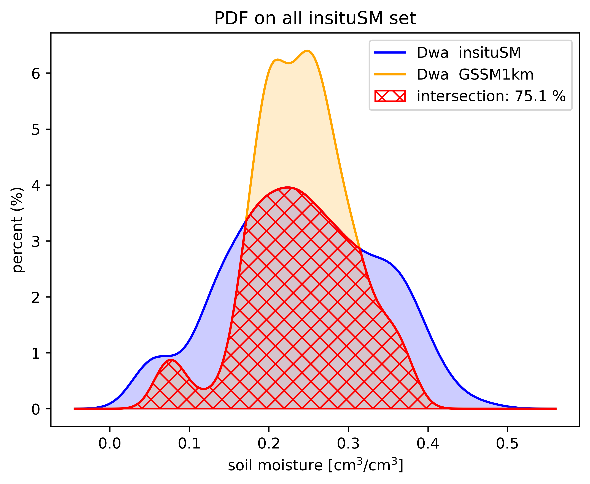

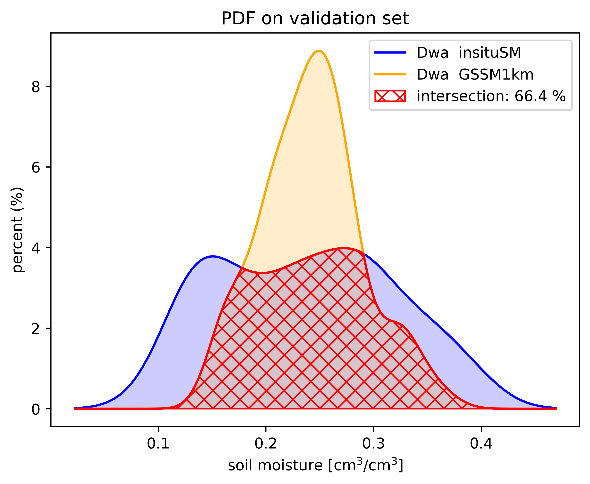

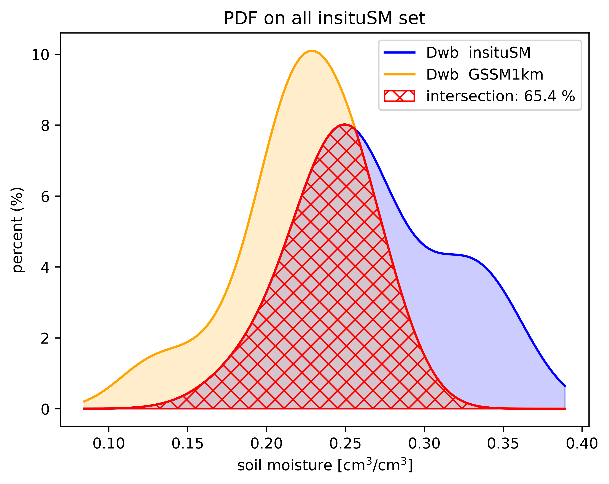

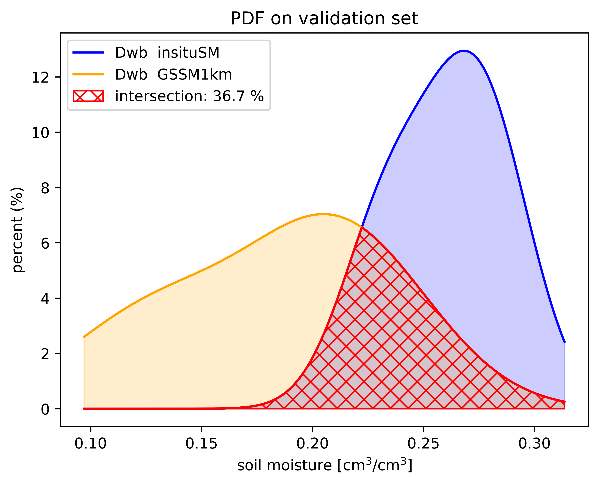

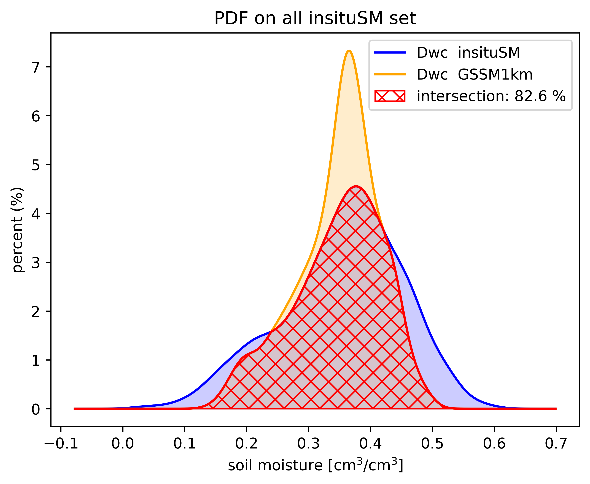

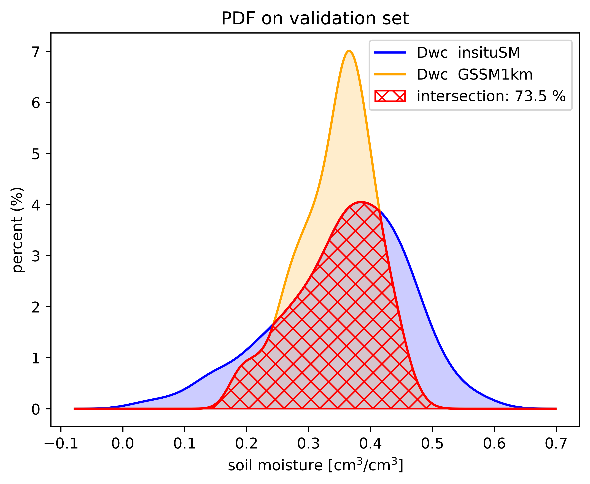

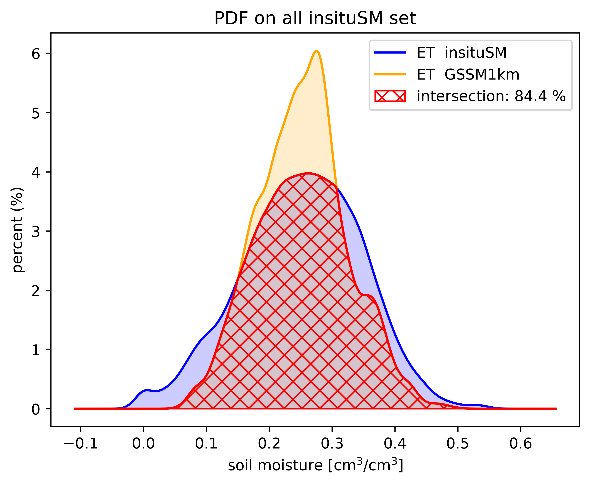

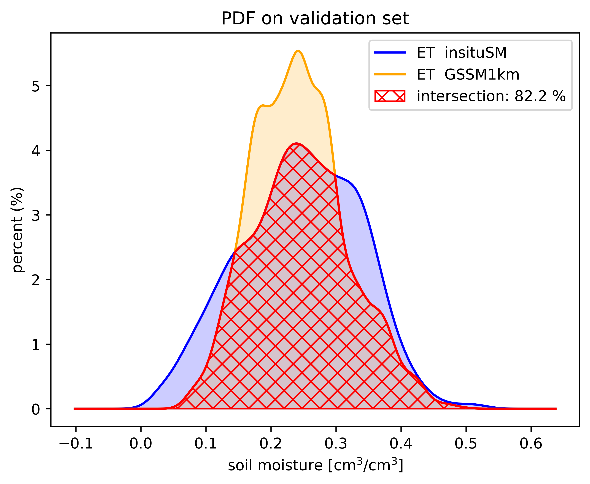


Fig. S3 PDF of insituSM and GSSM1km over 19 various climate zones at the global scale

There are 10 climate zones that do not have ground observations: Af, Am; Cwa, Cwc, Cfc, Csc; Dwd, Dfd, Dsa; EF.

For tropical climate zones (Af and Am), the GSSM1km is drier than other two datasets from the comparison with SMAP and ESACCI06.1. GSSM1km might be less skillful at predicting soil moisture in tropical regions, due to the sparse soil moisture stations in these regions.

For EF (Polar Eternal frost) area, it is eternal winter, with all 12 months of the year with average temperatures below 0 °C. It is mostly frozen, which is difficult to measure soil moisture (i.e. water in liquid form) with certainty ^21,22^.

For the 4 temperate (Cwa, Cwc, Cfc, Csc) and 3 continental (Dwd, Dfd, Dsa) climate zones, we do not have ground observations either. It is possible to have a higher uncertainty in these regions.

# 6 The latitudinal patterns

We calculated the latitudinal patterns among GSSM1km, ESACCI06.1 and SMAP in 2019 and 2020. The result shows the latitudinal pattern is consistent among three datasets in 2019 and 2020 (Fig. S4).


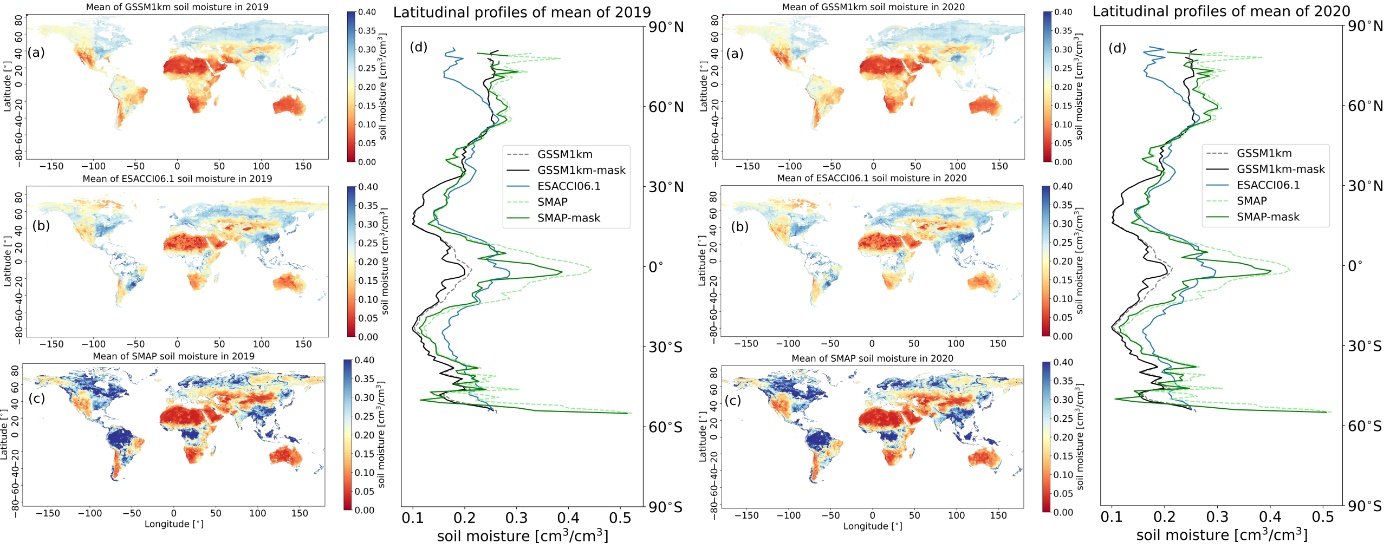


Fig. S4 Global mean SSM map of 2019 (left) and 2020 (right)

(a) GSSM1km; (b) ESA-CCI06.1; (c) SMAP. Areas in white means no data. (d) Comparison of latitudinal profiles among GSSM1km, GSSM1km-mask, ESA-CCI06.1, and SMAP, SMAP-mask. ESACCI06.1 is used as a mask for GSSM1km and SMAP because it has missing data.

# References

1 Khan, R. & Gilani, H. Global drought monitoring with big geospatial datasets using Google Earth Engine. *Environmental Science and Pollution Research* **28**, 17244-17264 (2021).

2 Muñoz-Sabater, J. *et al.* ERA5-Land: A state-of-the-art global reanalysis dataset for land applications. *Earth Syst. Sci. Data* **13**, 4349-4383 (2021).

3 Fan, Y., Li, H. & Miguez-Macho, G. Global patterns of groundwater table depth. *Science* **339**, 940-943 (2013).

4 Shangguan, W., Hengl, T., de Jesus, J. M., Yuan, H. & Dai, Y. Mapping the global depth to bedrock for land surface modeling. *J. Adv. Model. Earth Syst.* **9**, 65-88 (2017).

5 Hengl, T. *et al.* SoilGrids250m: Global gridded soil information based on machine learning. *PLoS one* **12**, e0169748 (2017).

6 Dorigo, W. *et al.* ESA CCI Soil Moisture for improved Earth system understanding: State-of-the art and future directions. *Remote Sens. Environ.* **203**, 185-215 (2017).

7 Xu, X. Evaluation of SMAP level 2, 3, and 4 soil moisture datasets over the Great Lakes region. *Remote Sens.* **12**, 3785 (2020).

8 Ali, S., Ghosh, N. & Singh, R. Rainfall–runoff simulation using a normalized antecedent precipitation index. *Hydrological Sciences Journal–Journal des Sciences Hydrologiques* **55**, 266-274 (2010).

9 Hillel, D. & Hatfield, J. L. *Encyclopedia of Soils in the Environment*. Vol. 3 (Elsevier Amsterdam, 2005).

10 Shiff, S., Helman, D. & Lensky, I. M. Worldwide continuous gap-filled MODIS land surface temperature dataset. *Sci. Data* **8**, 1-10 (2021).

11 Chen, J. *et al.* A simple method for reconstructing a high-quality NDVI time-series data set based on the Savitzky–Golay filter. *Remote Sens. Environ.* **91**, 332-344 (2004).

12 Kirkby, M. in *Process in physical and human geography* Ch. Hydrograph modeling strategies

69-90 (1975).

13 Beven, K. J. & Kirkby, M. J. A physically based, variable contributing area model of basin hydrology/Un modèle à base physique de zone d'appel variable de l'hydrologie du bassin versant. *Hydrol. Sci. J.* **24**, 43-69 (1979).

14 Pradhan, N., Tachikawa, Y. & Takara, K. A downscaling method of topographic index distribution for matching the scales of model application and parameter identification. *Hydrological Processes: An International Journal* **20**, 1385-1405 (2006).

15 Gruber, S. & Peckham, S. Land-surface parameters and objects in hydrology. *Developments in Soil Science* **33**, 171-194 (2009).

16 Yamazaki, D. *et al.* MERIT Hydro: A high‐resolution global hydrography map based on latest topography dataset. *Water Resources Research* **55**, 5053-5073 (2019).

17 Zhao, H., Zeng, Y., Lv, S. & Su, Z. Analysis of soil hydraulic and thermal properties for land surface modeling over the Tibetan Plateau. *Earth Syst. Sci. Data* **10**, 1031-1061 (2018).

18 Hillel, D. *Introduction to environmental soil physics*. (Elsevier, 2003).

19 Simons, G., Koster, R. & Droogers, P. (tech. rep. Report 213). FutureWater, 2020).

20 Entekhabi, D., Reichle, R. H., Koster, R. D. & Crow, W. T. Performance metrics for soil moisture retrievals and application requirements. *J. Hydrometeorol.* **11**, 832-840 (2010).

21 Mwangi, S., Zeng, Y., Montzka, C., Yu, L. & Su, Z. Assimilation of cosmic‐ray neutron counts for the estimation of soil ice content on the eastern Tibetan Plateau. *J. Geophys. Res. Atmos.* **125**, e2019JD031529 (2020).

22 Yu, L., Zeng, Y., Wen, J. & Su, Z. Liquid‐vapor‐air flow in the frozen soil. *J. Geophys. Res. Atmos.* **123**, 7393-7415 (2018).
